# DNA methylation landscapes of 1538 breast cancers reveal a replication-linked clock, epigenomic instability and *cis-*regulation

**DOI:** 10.1038/s41467-021-25661-w

**Published:** 2021-09-13

**Authors:** Rajbir Nath Batra, Aviezer Lifshitz, Ana Tufegdzic Vidakovic, Suet-Feung Chin, Ankita Sati-Batra, Stephen-John Sammut, Elena Provenzano, H. Raza Ali, Ali Dariush, Alejandra Bruna, Leigh Murphy, Arnie Purushotham, Ian Ellis, Andrew Green, Francine E. Garrett-Bakelman, Chris Mason, Ari Melnick, Samuel A. J. R. Aparicio, Oscar M. Rueda, Amos Tanay, Carlos Caldas

**Affiliations:** 1 Cancer Research UK Cambridge Institute, Li Ka Shing Centre, University of Cambridge, Cambridge, UK; 2 Department of Oncology, University of Cambridge, Cambridge, UK; 3 Department of Computer Science and Applied Mathematics, and Department of Biological Regulation, Weizmann Institute of Science, Rehovot, Israel; 4 Mechanisms of Transcription Laboratory, The Francis Crick Institute, London, UK; 5 Cancer Research UK Cambridge Centre, Cambridge, UK; 6 Cambridge Experimental Cancer Medicine Centre and NIHR Cambridge Biomedical Research Centre, Cambridge University Hospitals NHS Foundation Trust, Cambridge, UK; 7 Research Institute in Oncology and Hematology, Winnipeg, Manitoba Canada; 8 School of Cancer and Pharmaceutical Sciences, King’s College London, London, UK; 9 Division of Cancer and Stem Cells, School of Medicine, University of Nottingham and Nottingham University Hospital NHS Trust, Nottingham, UK; 10 Division of Hematology and Medical Oncology, Weill Cornell Medicine, New York, NY USA; 11 Department of Medicine, University of Virginia School of Medicine, Charlottesville, VA USA; 12 Department of Biochemistry and Molecular Genetics, University of Virginia School of Medicine, Charlottesville, VA USA; 13 Department of Physiology and Biophysics, Weill Cornell Medicine, New York, NY USA; 14 Department of Molecular Oncology, British Columbia Cancer Research Centre, Vancouver, British Columbia Canada; 15 Present Address: German Cancer Research Center (DKFZ), Heidelberg, 69120 Germany

**Keywords:** Breast cancer, Epigenomics

## Abstract

DNA methylation is aberrant in cancer, but the dynamics, regulatory role and clinical implications of such epigenetic changes are still poorly understood. Here, reduced representation bisulfite sequencing (RRBS) profiles of 1538 breast tumors and 244 normal breast tissues from the METABRIC cohort are reported, facilitating detailed analysis of DNA methylation within a rich context of genomic, transcriptional, and clinical data. Tumor methylation from immune and stromal signatures are deconvoluted leading to the discovery of a tumor replication-linked clock with genome-wide methylation loss in non-CpG island sites. Unexpectedly, methylation in most tumor CpG islands follows two replication-independent processes of gain (MG) or loss (ML) that we term epigenomic instability. Epigenomic instability is correlated with tumor grade and stage, *TP53* mutations and poorer prognosis. After controlling for these global trans-acting trends, as well as for X-linked dosage compensation effects, *cis*-specific methylation and expression correlations are uncovered at hundreds of promoters and over a thousand distal elements. Some of these targeted known tumor suppressors and oncogenes. In conclusion, this study demonstrates that global epigenetic instability can erode cancer methylomes and expose them to localized methylation aberrations *in-cis* resulting in transcriptional changes seen in tumors.

## Abstract (web summary)

Understanding the molecular mechanisms underlying DNA methylation in cancer and its clinical relevance remains crucial. Here, the authors study RRBS-based profiles of 1538 breast tumours and 244 normal breast tissues from the METABRIC cohort and report epigenomic instability and cis-regulatory effects.

## Introduction

Cancer cells display a combination of altered gene regulatory programs that endow them with capabilities for growth, stromal interactions, immune evasion, and metastasis^1^. The role of somatic mutations in driving carcinogenesis is established, even though most mutations are passengers. The regulatory state of cancer cells is also known to be correlated with, and in many cases driven by, multiple additional layers of aberrant epigenetic controls. DNA methylation is the most extensively characterized, and the landscape of cancer genome methylation has been analyzed in many tumor types, to date mostly using microarrays^2,3^. It has long been known that globally tumors lose methylation^4^ and also that normally unmethylated CpG islands can become methylated in cancer resulting in gene repression^5^. Despite these efforts, the forces that drive pervasive methylation changes in tumors, and the impacts of methylation aberration on tumorigenesis are still poorly understood. Furthermore, how tumor epigenetics affect clinical outcomes and response to treatment remains unclear.

The genomic landscapes of breast tumors are dominated by copy number aberrations (CNA)^6^ and a few genes hit by somatic mutations in a large fraction of cases (*TP53* in 35.4%, *PIK3CA* in 40.1%)^7^. This makes it challenging to understand disease progression and management based on genetic profiling alone^8^. On the other hand, bulk transcriptional profiling of tumors is in routine clinical use to assist in prognostication and treatment optimization, indicating that breast cancers recurrently converge onto common molecular states even when the potential drivers (genetics and others) are diverse^9^. In search for non-genetic drivers, early analysis of DNA methylation focused on hypermethylation of CpG islands of key driver genes, including estrogen receptor^10^ and *BRCA1*^11^. The initial hypotheses that such focused epigenetic aberrations played a driver role has been questioned as methylation profiling scaled in coverage and accuracy^12^. Indeed CpG island hypermethylation was found in thousands of genes in breast cancer^13^, indicating that promoter methylation may instead represent a global trend of loss of protection from methylation accumulation^14^. Trends of hypomethylation throughout large genome territories are also observed when analysis is extended from CpG islands toward the entire genome^15,16^, further suggesting one or more global processes are driving pervasive methylation change in cancer.

To understand the potential driver roles of mutational, transcriptional, and epigenetic aberrations in breast cancer, a comprehensive and systematic strategy that puts each regulatory layer in the context of other layers is needed. For analysis of DNA methylation, the major challenge is to understand which methylation changes are part of global epigenetic remodeling trends, what are the processes (genetic, transcriptional, and other) regulating such trends, and which loci are affected by methylation changes *in cis* that cannot be explained by global trends and hence may have a direct regulatory effect (i.e., being a driver event).

The METABRIC cohort includes over 2000 breast tumor samples that were previously characterized extensively clinically, genetically, and transcriptionally. To this rich resource, we now add data on DNA methylation landscapes using reduced representation bisulfite sequencing (RRBS). This leads to several conclusions that we now propose in a unified model that accommodates multiple previous lines of evidence of breast cancer DNA methylation. The model represents 6 global trends that affect breast DNA methylation profiles, two involving contamination of immune and stromal cells, one representing replication-linked hypomethylation clock, one involving X-chromosome dosage compensation, and two representing epigenetic instability at CpG islands. Based on this model, we demonstrate methylation in hundreds of promoters and thousands of distal elements to be correlated with gene expression specifically *in cis*, highlighting the important role of the global methylation trends in providing the basis for numerous transcriptional aberrations, including the classical *BRCA1* hypermethylation effect.

## Results

### Methylation profiling of the METABRIC cohort

**Fig. 1:**
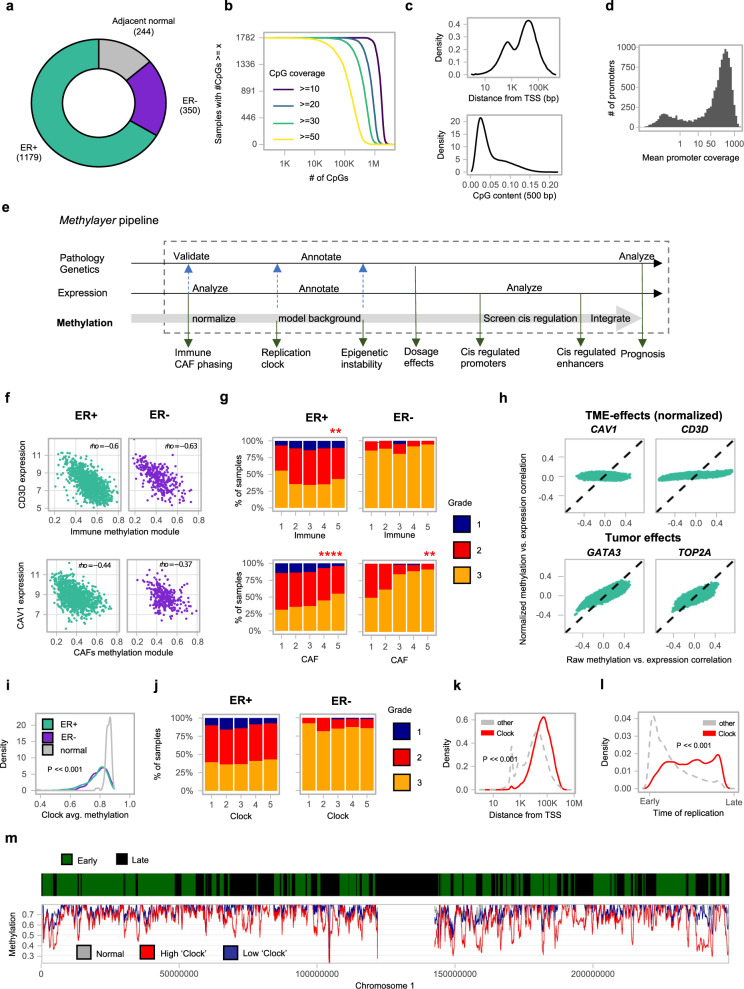
Dissecting tumor, immune, and CAF methylation in the METABRIC cohort. **a** Distribution of METABRIC samples used for RRBS profiling. **b** Number of samples (*Y* axis) with a given number of CpGs (*X* axis) covered with at least 10, 20, 30, or 50 reads. For example, in all samples 449,710 CpGs are covered with over 10 reads. **c** Distribution of TSS distance (top) and CpG content (bottom) for CpGs covered by at least 5 reads in half or more samples. **d** Distribution of mean promoter coverage over all METABRIC samples, considering 13,198 active promoters (“Methods”). **e** The *Methylayer* analysis pipeline. Integration of data is marked by black arrows. Annotation of the model is marked by blue dashed arrows. **f** Correlation of average expression of *CD3D/CAV1* and the immune/CAF methylation module. **g** Distribution of tumor grade stratified by five bins of Immune/CAF methylation scores (*χ*^2^ test: *p* = 0.0056 for Immune score in ER+, *p* = 0.3052 for Immune score in ER−, *p* = 0.00001 for CAF score in ER+, *p* = 0.0031 for CAF score in ER−). *p ≤ 0.05, **p ≤ 0.01, ***p ≤ 0.001, ****p ≤ 0.0001. **h** Distribution of correlations of four key gene expression profiles with raw-methylation levels of individual gene promoters (*X* axis), versus the correlation derived after normalizing methylation for CAF/immune composition using a K-nn strategy (as described in “Methods”). Flattening of the post normalization correlations demonstrate the effect of the CAF/immune normalization, while maintenance of the post normalization correlations demonstrates that the genes that are not affected by normalization. **i** Distribution of average (non-normalized) methylation over loss-clock loci (correlation over 0.6 with the score) for normal breast, ER+ and ER− breast cancer samples (two-tailed Kolmogorov–Smirnoff test, *p* < 2.2e−16). **j** Distribution of tumor grade stratified by five bins of loss-clock score. **k** Distribution of TSS distance for loss-clock loci versus other loci (two-tailed Kolmogorov–Smirnoff test, *p* < 2.2e−16). **l** Distribution of time-of-replication for loss-clock loci (red) versus overall distribution of non-promoter loci (gray) (two-tailed Kolmogorov–Smirnoff test, *p* < 2.2e−16). **m** Replication time classifications for chromosome 1 (top color-coded bar). The average methylation in non-promoter loci, computed for normal breast tissues (gray), and two ER+ breast cancer groups with high (red) and low (blue) clock scores, respectively, is shown below.

The METABRIC cohort allowed us to analyze methylation trends within the clinical, genomic, and transcriptional data available for 1538 tumor samples and 244 adjacent normal tissues (Fig. 1a, Extended Data Fig. 1a, Supplementary Data 1). We tuned the RRBS approach to cover on average 3.3 M CpGs with over 1 read per sample (Extended Data Fig. 1b). Overall, we used 30.4B reads from 1782 breast tumor and normal samples to cover a broad genomic distribution of loci and facilitate analysis of both global methylation trends as well as local dynamics of methylation in regulatory elements and promoters. Using our version of the RRBS protocol 93% of the samples were covered by more than 10 reads for more than 1 M CpGs (Fig. 1b). Only 9% of the reads mapped to bona-fide promoters (Fig. 1c), providing extensive sampling of non-promoter loci with medium and low CpG density, and enhancers in particular (Extended Data Fig. 1c, d). 75% of the promoters were covered with over 20 reads on average (mean coverage 246), facilitating quantitative analysis downstream (Fig. 1d).

### Layered modeling of breast tumor methylation

A major challenge in the understanding of tumor methylation is the convergence of multiple mechanisms, dynamics, and biases onto complex genome-wide methylation profiles. Using the comprehensive and multifaceted METABRIC dataset as a working model, we developed a semi-supervised computational strategy (*Methylayer*) for layered modeling of tumor methylation dynamics (Fig. 1e). The principle underlying *Methylayer* relies on integration of gene expression, genetics, and clinical information for computational peeling of confounders (tumor microenvironment [TME] effects), and then inference of global trends that can stochastically affect all or almost all of the methylome, in particular due to replication age and copy number aberration effects (see “Methods” for details). Based on this top-down approach, *Methylayer* can robustly screen for candidates for epigenetic *cis*-regulation, and derive prognostic metrics. We applied *Methylayer* to ER+ and ER− METABRIC samples separately and compared dynamics in the two classes of tumors. We validated the robustness of the approach using unsupervised non-negative matrix factorization (Supplementary note 1), and by applying the pipeline to an independent TCGA breast cancer dataset (Supplementary note 2) with highly reproducible results to those reported below.

As shown in Fig. 1f, integration of gene expression allows *Methylayer* to identify TME effects as major data confounders diversifying methylation landscapes acquired from tumor biopsies. The algorithm detected a strong immune signature in cross-correlation of gene expression and promoter methylation data (Extended Data Fig. 2a–c), unambiguously anchored by expression profiles of many marker genes (*CD3*, *CD8*) and checkpoints (*CTLA4*, *PD-1*) (Supplementary Data 2). In parallel, a cancer-associated fibroblast (CAF) signature was anchored by *FAP*, *CAV1*, *VIM*, and additional genes (Supplementary Data 3). TME signatures were correlated with tumor grade (Fig. 1g) and were validated by independent deconvoluted expression profiles and pathological metrics (Extended Data Fig. 2d–g). Following inference of TME signatures, we applied a novel K-nn normalization algorithm (“Methods”, Fig. 1h) that provided *Methylayer* substantially reduced TME bias when inferring tumor methylation layers downstream (Extended Data Fig. 3).

### A replication-linked methylation clock process correlates with pervasive loss of methylation in tumors

*Methylayer* clustering of TME-normalized methylation next identified a highly correlated group of CpGs (Extended Data Fig. 4a, b) that together span a range of reduced methylation levels (mean methylation = 0.78 ± 0.15 in ER+, 0.79 ± 0.13 in ER−) in tumors compared to normal controls (Fig. 1i). This methylation layer did not correlate with tumor grade (Fig. 1j) and was denoted as the *clock layer* for reasons discussed next. Clock layer CpGs showed low CpG content and although associated with distal localization in relation to promoters (Fig. 1k), were under-represented at putative regulatory elements (based on histone modifications) (Extended Data Fig. 4c, d). Genomes replicate in S-phase through a regulated process defining early and late replication domains^17,18^. Interestingly, reduction in tumor methylation of the clock layer was much more intense in domains replicating late in S (Fig. 1l, m, Extended Data Fig. 4e–h). This is consistent with previous reports suggesting loss of DNA methylation in aging and cancer can be linked with accumulation of methylation errors (“epi-mutations”) that are correlated with the replication process^19–21^. Screening gene expression signatures across METABRIC did not uncover pervasive transcriptional programs linked with the methylation clock layer. The few genes that were significantly correlated with the global clock trends were enriched for cancer-testis antigen (CTA) (Extended Data Fig. 5a) and were showing predisposition for infrequent de-repression in tumors with advanced methylation loss (Extended Data Fig. 5b), consistent with recent analysis of clonal methylation and expression in vitro^22^. No parallel replication-linked methylation gain process was observed. Furthermore, linkage of tumor replication loss clock and existing methylation age clocks was limited (Extended Data Fig. 5c–g). In summary, these data taken together suggest pervasive dynamics of methylation loss clock in cancers that is strongly linked to genome replication trends, with low impact on gene expression signatures, but with potential linkage to CTA de-repression.

### The epigenomic instability signatures

**Fig. 2:**
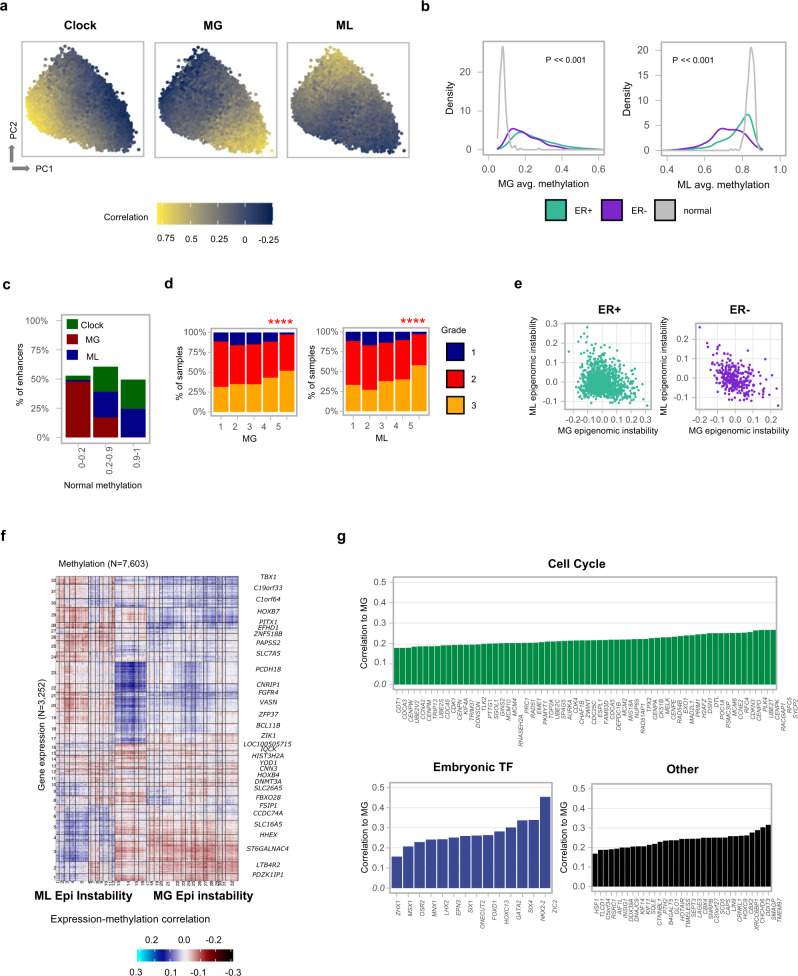
Epigenomic instability in breast cancers. **a** Color-coded maps represent 201,082 genomic loci projected over the two first principal components given their correlations with the three *Methylayer* scores. **b** Distribution of average (non-normalized) methylation over MG and ML loci (correlation over 0.5 with each score) for normal breast, ER+ and ER− breast cancer samples (two-tailed Kolmogorov–Smirnoff test: MG loci, *p* < 2.2e−16; ML loci, *p* < 2.2e−16). **c** Fraction of enhancers (grouped by their normal methylation level) that are linked (correlation > 0.25) with each of the three *Methylayer* scores. **d** Distribution of ER+ tumor grade stratified by five bins of MG/ML methylation scores. ****p < 0.0001 (*χ*^2^ test: MG loci, *p* = 0.000002; ML loci, *p* < 5.4e−10). **e** Comparing ML and MG scores over ER+ and ER− samples. **f** Clustered correlation heat map between normalized methylation profiles (columns, including all loci with correlation >0.3 for MG or ML) and matching gene expression (rows) in ER+ tumors. Clusters are labeled by their top correlated gene. Complete information is available in Supplementary Data 4. **g** Groups of genes showing positive expression correlation with the MG score (see Supplementary Data 6. Complete information for ML score is also available in this table).

*Methylayer* analysis identified two further global methylation layers with remarkably different characteristics (Fig. 2a, Extended Data Fig. 6a, b). The first of these layers is denoted as the epigenomic instability methylation gain (MG) layer, involving CpGs that are unmethylated in normal tissues (Fig. 2b), but show a spectrum of hypermethylation in tumors. Remarkably, 45% of the sampled intermediate-high CpG content enhancers show strong correlation with the MG layer (Fig. 2c, Ext Data Fig. 6c), as well as 2995 of the promoters in our data. A much smaller fraction of the CpG sites is part of the epigenetic instability methylation loss (ML) layer, which is partially methylated in normal tissues, but shows a spectrum of reduced methylation in tumors. MG CpGs are enriched in low CpG content enhancers. Remarkably, quantifying MG and ML methylation layers across METABRIC (where high scores indicate larger differences from normal tissues) showed grade-dependent distributions in ER+ tumors (Fig. 2d, Extended Data Fig. 6d). Furthermore, MG layer methylation was positively correlated with the expression of a large number of genes *in trans* (Fig. 2f, Supplementary Data 6). Most notably, these include genes linked to mitotic spindle assembly and regulation, DNA damage repair, DNA replication, several embryonic development homeobox transcription factors (for example, the early mesenchymal factor *Msx1*), calcium signaling, and sterol metabolism genes (Fig. 2g, Supplementary Data 6).

We adapted our previously developed methodology^14^ to analyze epi-polymorphism at the MG and ML loci (Extended Data Fig. 7a, b) indicating methylation heterogeneity at these loci is as high as the global background trend, and showing that accumulation of methylation is likely the outcome of multiple stochastic events rather than takeover of specific epi-alleles. Further analysis showed MG and ML scores varied extensively across the Integrative clusters^6^, which stratify breast cancers into genomic copy number driver-based subtypes. High epigenomic instability scores were observed for IntClust 1, 2, 5, 6, and 9 (Extended Data Fig. 7c). Interestingly these are the ER+ tumor subtypes we recently showed tend to have higher incidence of later disease relapse^8^.

In summary, these analyses show that in addition to the replication loss clock, a large number of loci are affected by a process of methylation gain (MG) and loss (ML), and that this process, rather than the methylation loss clock, is linked with tumor progression, genomic subtypes, and tumor gene expression state.

### Methylation and expression are linked *in cis* at hundreds of promoters and distal elements

**Fig. 3:**
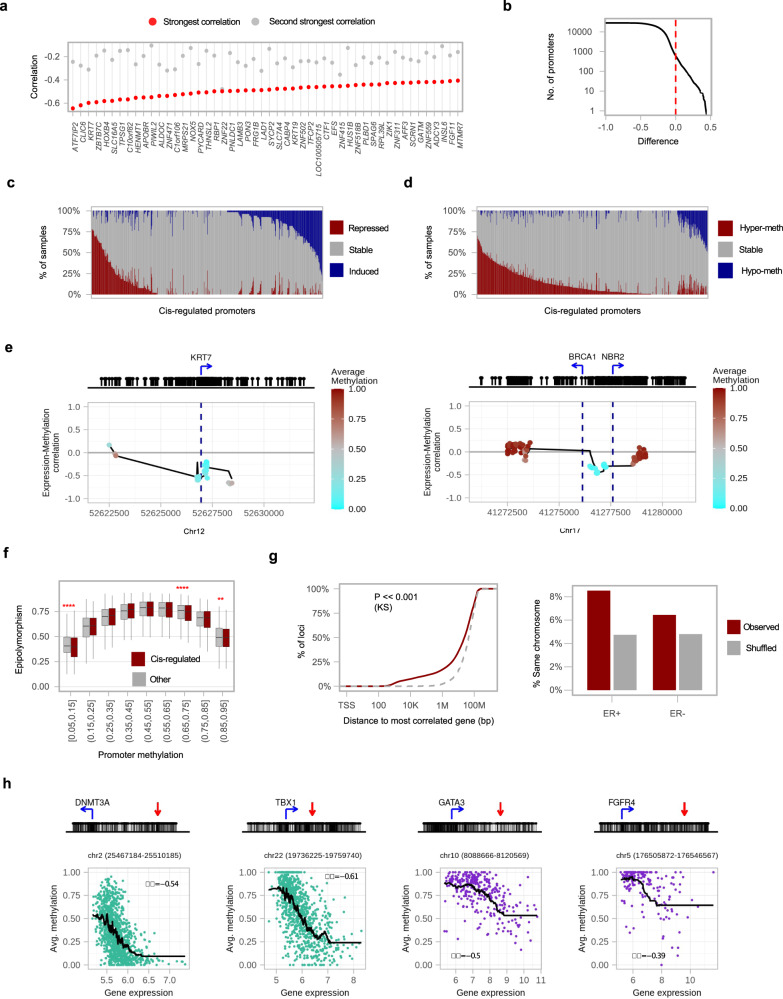
Expression–methylation correlation *in cis*. **a** Plot of the 50 genes with strongest negative correlation (red) of expression with their own promoter methylation profile (*in cis* E–M correlation) in ER+ tumors, compared to the correlation with the second strongest promotor locus in the genome (out of 9360 candidates, gray). **b** Distribution of the difference between *in cis* E–M correlation, and the top correlation of the same gene with any other promoter. Positive values represent cases where the *in cis* E–M correlation is the maximum. **c** For 612 genes with support for *in cis* E–M correlation, we show the distribution of differential tumor expression relative to matched normal tissues. Repressed/induced: over twofold change. **d** For 612 promoters with support for *in cis* E–M correlation, we show the distribution of differential methylation compared to matched normal tissues. Hyper-/hypo-methylated: over 0.2 different in average methylation. **e** Correlation of CpG methylation with gene expression in *KRT7* locus (in ER+ tumors) and *BRCA1* (in ER− tumors). **f** Distribution of Epi-polymorphism for promoters defined with high *in cis* E–M correlation. Shown are promoters that had at least one tumor sample with average methylation above 0.05 (red, *n* = 306). Loci are grouped by average promoter methylation and other promoter loci (gray, *n* = 3570) are provided for control. *p ≤ 0.05, **p ≤ 0.01, ***p ≤ 0.001, ****p ≤ 0.0001 (one-sided Wilcox test). The middle line indicates the median, box l*i*mits represent quartiles, and whiskers are 1.5× the interquartile range. **g** Left: Cumulative distribution of the distance between methylation loci and the promoter with highest expression correlation to them (within the same chromosome, two-tailed Kolmogorov–Smirnoff test, *p* < 2.2e−16). Right: fraction of loci for which the best-correlated promoter is located on the same chromosome. Gray line/bars represent shuffled controls (see “Methods”). **h** Examples for matching expression and methylation for non-promoter genomic loci located in the proximity of their most correlated gene (*DNMT3A* and *TBX1* in ER+ tumors; *GATA3* and *FGFR4* in ER− tumors). Above: Location of the non-promoter genomic loci (red arrow) relative to the TSS of the gene (blue arrow). Below: Correlation of the matching expression of the most correlated gene (*X* axis) and methylation (*Y* axis) for the non-promoter genomic loci.

The MG/ ML epigenomic instability layers we defined above correlate transcriptional and epigenetic changes for a large number of loci. Specific methylation-expression regulatory relationships can be supported when a promoter methylation signature correlates with its matching gene expression signature (*in cis*) at significantly higher levels than those predicted by the above epigenomic instability effects working *in trans*. We screened for such scenarios by comparing correlation between promoter methylation with its own expression to its correlation with any of the other 9360 gene expression profiles. Applying this strategy systematically and comparing to shuffled controls (see “Methods”), we identified 423 promoters in ER+ and 185 in ER− (FDR < 0.01; 1053 in ER+ and 448 in ER− if increasing FDR to <0.05) that are likely to regulate expression by their *in cis* methylation (Fig. 3a, b, Supplementary Data 8). These promoters have low, but non-zero methylation in normal tissues (15 ± 22%) weakly increasing overall in tumors on average (20 ± 21%) (Extended Data Fig. 8a). Overall, 34%, 50%, and 16% of these loci were located, respectively, in high (>8%), intermediate (>4%), and low (≤4%) CpG content regions (Extended Data Fig. 8b). *In cis* methylation-expression correlation was usually (but not exclusively) observed for genes showing both repression and induction in tumors compared to normal, in association with their hypermethylation (Fig. 3c, d). To find which of these localized and specific *cis*-regulated genes are part of larger co-regulated gene modules (Fig. 3e), we computed correlation of gene expression profiles (Extended Data Fig. 8c) and confirmed most genes in the group were not part of larger expression clusters (392 out of 423 with best correlation partner at *r* < 0.5). These data suggest the existence of hundreds of distinct regulatory “logics” which are generally specific to individual genes and may act as drivers of inter-tumor expression variation for these genes, independently of the global methylation loss clock and epigenomic instability trends we quantified above. Remarkably, analysis of epi-polymorphism in these promoters supported significantly reduced methylation diversity specifically when methylation average is low (Fig. 3f, *P* ≪ 0.001 for lower epi-polymorphism at these sites), suggestive of epigenetic convergence, or even selection at these loci during tumorigenesis.

To search for non-promoter *in cis* regulators, we identified for each methylation locus its most strongly correlated gene expression profile, and selected those loci in which this best correlation gene partner (i.e., its TSS) was located within the immediate chromosomal domain (Fig. 3g, h, Supplementary Data 8). This analysis identified 2680 distal *cis*-elements in ER+ and 1332 in ER− (best partner, FDR < 0.1), out of which 67% were located within 50 kb of the promoter, and 34% from 50 to 500 kb from the promoter. As with promoters, analysis of epi-polymorphism in these loci supported significantly reduce methylation diversity, again suggesting convergence/ selection (Extended Data Fig. 8d). Motif analysis and comparison with ChIP-seq profiles confirmed that these elements are not linked with one dominating regulatory mechanism, further suggesting methylation changes are driven *in cis* for these loci, rather than being regulated by a common mechanism *in trans*.

In summary, we show that while the genome-wide methylation loss clock and epigenomic instability affect nearly all CpGs in the genome in a coordinated fashion, localized methylation of promoter and distal elements correlates and perhaps regulates specifically hundreds of genes *in cis*. Classical hypothesized scenarios for epigenetic gene repression as driving tumorigenesis can now be rationalized within a rich background model. For example, *BRCA1* promoter methylation is correlated strongly and specifically with *BRCA1* gene expression in ER− tumors and *KRT7* promoter methylation with *KRT7* gene expression in ER+ tumors (Fig. 3e, Extended Data Fig. 8e). We have recently reported differential KRT7 protein expression in luminal cell populations associates with survival^23^. While it is likely that many cases of *in cis* methylation/expression correlation will have a passenger effect, just like most somatic mutations, it is also evident that some *cis*-regulated genes participate in tumorigenesis in a way analogous to *BRCA1*.

### X-linked dosage compensation in tumors

DNA methylation and gene expression linkage can be further modulated in tumor cells with copy number aberrations (CNA). The asymmetric inactivation of the X chromosomes serves as a classical model for such interplay^24,25^. We identified a cluster of X-linked promoters whose methylation profiles show specific correlation with a gene module including the XIST noncoding RNA and additional X-linked genes (Extended Data Fig. 9a, b, Supplementary Data 4) suggesting that they are involved in the maintenance of X methylation and its re-establishment following X-chromosome CNA. Next, we investigated localized dosage compensation through methylation on loci within the X chromosome. In ER− tumors we observed ~50% and ~25% X-chromosome partial loss and gain, respectively, where in ER+ we observed ~20% partial loss, and a low (<10%) rate of chromosome gain events (Extended Data Fig. 9c). In ER- tumors, we observed methylation to decrease when copy number decreased from 2 to 1 and increase when copy number increase from 2 to 3, suggesting one copy of active and unmethylated X per locus is maintained even after X-chromosome aberrations (Extended Data Fig. 9d, e). A similar observation was made for ER+ loss events (but not in the relatively rare 2N to 3N gain events). Importantly, the gene expression emitted from the X-linked promoters associated with CNA was not scaling with copy number (Extended Data Fig. 9f, i), suggesting dosage compensation is strongly correlated with the methylation changes we observed (however, we did observe increased expression in ER+ 2N to 3 N gain loci, *P* ≪ 0.001, KS test, Extended Data Fig. 9i).

Similar analysis applied to autosomes showed most loci showed no scaling of methylation with copy number (Extended Data Fig. 9g, h), but a small number of loci showed increase in methylation in 3N vs 2N gain loci (279 genes in ER+, 197 genes in ER−) or in 4N vs 2N amplification loci (521 genes in ER+, 260 genes in ER−). In addition, 26 genes in ER+ and 20 genes in ER− showed decreased methylation in 1N vs 2N (Supplementary Data 9). Importantly, loci gaining or losing autosome copy number showed a strong dosage effect (Extended Data Fig. 9i), but this effect was almost eliminated when restricting analysis to loci with compensated methylation differential (methylation decrease in 2N to 1N, increase in 2N to 3N). For instance, dosage associated increased methylation was associated with reduced *PROM1* expression in ER+ tumors with 3N copies; and with reduced *SOX1* expression in ER− tumors with 3N copies.

In summary, X inactivation constitutes a powerful dosage compensation engine, and methylation of gained X-linked loci in ER− tumors may be driven by the X inactivation apparatus. In non-X-linked loci, localized dosage compensation can be facilitated through methylation in a more restricted class of genes such as *PROM1* and *SOX1*.

### Methylation landscapes of breast cancers are correlated with genomic aberrations and are predictive of survival

**Fig. 4:**
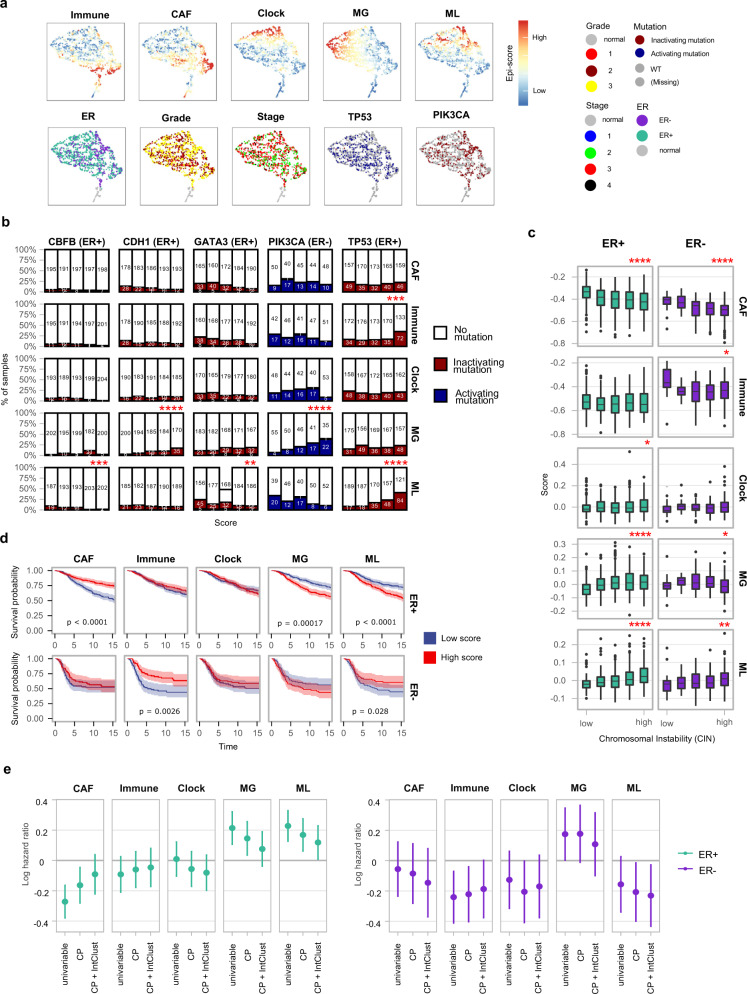
Epigenomic instability correlates with genomic features and with poor survival. **a** Projection of METABRIC tumor samples on a unified epigenetic signatures space, colored by the five epigenetic scores, ER status, grade, stage, *TP53*, and *PIK3CA* mutations. **b** Tumors were stratified into five groups (bars) for each of five different methylation signatures (rows). Shown are the fraction of cases with specific mutations in each stratum. *FDR ≤ 0.05, **FDR ≤ 0.01, ***FDR ≤ 0.001, ****FDR ≤ 0.0001 (two-sided Wilcox test). **c** Boxplots show distribution of epigenomic signatures in ER+ (left, *n* = 1108) and ER− (right, *n* = 310) cancers stratified according to estimated chromosomal instability levels (derived from CNA data). *p ≤ 0.05, **p ≤ 0.01, ***p ≤ 0.001, ****p ≤ 0.0001 (Spearman *rho*). The middle line indicates the median, box limits represent quartiles, and whiskers are 1.5× the interquartile range. **d** Kaplan–Meier survival plots for ER+ (top, *n* = 1108) and ER− (bottom, *n* = 310) tumors grouped into high-scoring and low-scoring groups for each epigenomic signature (top 1/3 and bottom 1/3 of the samples). 95% confidence intervals are shown. Log-rank *p*-values for survival difference are reported. **e** Log hazard ratios (normalized by SD) calculated for each epigenomic signature using 4 distinct Cox proportional hazards regression models: (i) Univariable (unadjusted for confounders); CP (adjusted for clinico-pathological variables—age, grade, tumor size, and lymph node status); CP + IntClust (adjusted for clinico-pathological variables and integrative cluster subtypes). Censoring at 15 years. Mean with 95% confidence intervals are shown. Models are stratified for ER+ (left, *n* = 1055) and ER− (right, *n* = 300) tumors.

Projection of overall methylation landscapes in 2D across samples using UMAP highlighted the combinations of epigenetic signatures shaping all breast tumors and normal samples (Fig. 4a). To integrate with driving genetic events, we screened the METABRIC genomic data for association with the five different epigenetic scores. For each epigenetic score, we stratified tumors into five bins and estimated within each bin the penetrance of 171 SNVs (Fig. 4b) and Integrative clusters (Extended Data Fig. 10a). We detected highly significant correlations with *TP53*, *PIK3CA*, *CDH1, GATA3*, and *CBFB* SNVs (Fig. 4b), and to a lesser degree with mutation intra-tumor heterogeneity (Extended Data Fig. 10b). Most notably, *TP53* mutations were strongly linked with increased ML score (*P* ≪ 0.001, FDR corrected Wilcox test), and higher immune signature (*P* ≪ 0.001). Higher epigenetic instability was linked with *CDH1* mutation (*P* ≪ 0.001) in ER+ tumors and *PIK3CA* mutations in ER− (*P* ≪ 0.001). Lower ML score was associated with *CBFB* and *GATA3* mutations (*P* < 0.001) in ER+ tumors. Together this analysis linked the different methylation scores with distinct somatic genomic aberrations in breast cancers, and strongly implicated p53 somatic mutation with the loss of methylation in CpG islands that together constitute the ML score. Similar analysis linked higher epigenetic instability with higher chromosomal instability (CIN) in ER+ tumors (Fig. 4c). Significant correlations of the epigenetic signatures were also detected with driver CNAs (Extended Data Fig. 10c). In particular, *BRCA1* loss in ER+ tumors was strongly associated with increased ML and MG scores (*P* < 0.01 for MG, *P* < 0.001 for ML, FDR corrected Wilcox test), and gain of MYC was associated with increased MG and ML scores (*P* ≪ 0.001).

To evaluate the clinical impact of the epigenetic signatures, we analyzed patient survival stratified by epigenetic scores (Fig. 4d). This showed that high MG epigenetic instability was predictive of poor survival. Five-year disease-specific survival decreased from 91% (64%) to 83% (55%) for ER+ and ER− tumors, respectively (*P* ≪ 0.01 for ER+). ML epigenetic instability also correlated with poor survival in ER+ tumors and this remained significant even when excluding *TP53* mutant tumors from the analysis (Extended Data Fig. 10d). In contrast, the replication-linked loss clock showed no association with survival. We next performed Cox proportional hazard analysis, demonstrating the 15-year prognostic value of the epigenetic instability scores even when considering clinical, genetic, and transcriptional scores on the background (Fig. 4e). Finally, we exemplified the contribution of the new epigenetic scores to the multistate breast cancer progression model we recently reported^8^ (Extended Data Fig. 10e).

In summary, we showed epigenetic signatures, and in particular epigenetic instability are correlated with genomic features of tumors and predictive of disease stage and progression and that this holds true even when considering clinical, genetic, and transcriptional metrics. We hypothesize methylation scores provide a window into the tumor state which is not always reflected by other metrics.

## Discussion

**Fig. 5:**
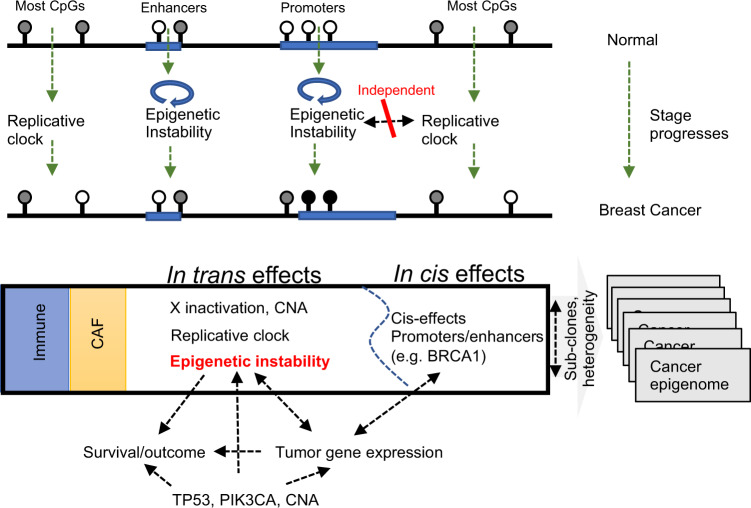
Unified model delineating the multi-factorial processes giving rise to breast cancer DNA methylation. As the carcinogenesis process progresses (top), epigenomes are affected by replication-dependent methylation loss in most of the genome, and by a second, uncorrelated epigenetic instability process modulating methylation in promoters and enhancers. When cancer epigenomes are surveyed (middle), the observed profiles involve a superposition of TME signatures, with the patient-specific replication and instability signatures, and with epigenetic dosage compensation. These processes are each affecting a large number of genomic loci through one common mechanism (*in trans* effects). Additional localized patient-specific methylation aberrations are uncorrelated with these *in trans* effects and may regulate gene expression *in cis*. Deconvolution of these multi-layered effects shows linkage between epigenetic instability and disease stage and prognosis (bottom). Cancer epigenomic heterogeneity is also induced by cellular heterogeneity (such as clonal structure).

We profiled DNA methylation across 1538 METABRIC breast tumors and performed large-scale integrative analysis of their methylation dynamics in transcriptional, genetic, and clinical contexts. The breadth, scope, and multifaceted nature of the METABRIC cohort were crucial in the delineation of the multi-factorial processes giving rise to breast cancer DNA methylation. This resulted in a model that can unify decades of conflicting evidence on passenger and driver roles for epigenetic changes in breast cancer, but also other tumor types (Fig. 5). We believe this model is a major contribution to understanding the impact of epigenetics on disease evaluation and management, and to therapeutic strategies combining modulation of epigenomic alterations with direct targeting of aberrant genes.

Cancer genomics studies reveal that tumors are frequently driven by a few key driver mutations, but that tumor evolution involves further adaptation and transformation that cannot be explained by canonical and recurrent genetics, while still converging on highly recurrent molecular and pathological outcome. Epigenetics was argued over the years to supplement tumor evolution with an alternative mechanism for silencing tumor suppressors (e.g., BRCA1), but its overall impact on tumors has remained controversial. We propose that epigenetic instability is an emerging hallmark of breast cancer, and most probably other tumor types. Much like genomic instability, epigenetic instability is not directly driving tumors—but its pervasive effect is predisposing tumor cells to develop regulatory plasticity and underlie the emerging activation of highly detrimental transcriptional programs, including de-differentiation (de-repression of embryonic TFs) and *trans*-differentiation (EMT genes). It was impossible to argue this before—due to the complexity of tumors and the multiple layers of regulation they gradually disrupt during malignant transformation.

The methylation loss-clock process can be hypothesized to represent another aspect of the widely acknowledged methylation ageing clock^26^. However, the loss clock we defined is quantifying methylation loss in low CpG content sites rather than the ageing clock’s methylation gain in CpG islands, and in both tumors and normal breast tissues, the loss signature is not correlated to patient age and may represent tumor cell replicative age instead. It is more difficult to understand the mechanisms underlying the epigenetic instability trends. We can hypothesize that the predisposition of tumors to lose protection against CpG islands methylation is linked to broad induction of multiple embryonic transcription factors and epigenetic complexes (in particular the polycomb machinery^27^). Several candidate genes are identified showing strong gene expression correlation to epigenetic instability across hundreds of tumors, suggesting avenues for mechanistic follow-up. It remains to be seen how such regulation affects tumor subtypes, or sub-clonal structure within tumors.

Each of the broad trends of methylation aberration we defined above is likely to be initially a consequence of the carcinogenic process. Nevertheless, we also show the cumulative effect of a very large number of epigenetic perturbations to be correlated specifically and *in cis* with hundreds of additional transcriptional changes. Such correlation is observed when expression and promoter/enhancer methylation changes are separated from and linked significantly beyond the broad global *in trans* trends. This may suggest epigenomic instability predisposes tumors to greater regulatory variation and flexibility, in a way resembling the impact of genomic instability on tumors. Breast tumors are fundamentally driven by the convergent effect of multiple genetic and other aberrations, few of which are appearing with very high penetrance. The discovery that epigenomic instability is pervasively observed in high-grade tumors, with prognostic power that is synergistic to clinical and genetic markers, may further hint toward its possible functional impact.

## Methods

### Patients and samples

For DNA methylation profiling, we sequenced 1782 samples: 1538 primary breast tumors and 244 adjacent normal samples. A total 1418 tumors were from the 1980 included in the original METABRIC report^6^. The additional 120 tumors were part of the METABRIC cohort but these samples either failed quality checks on the platforms used at the time, lacked corresponding gene expression data, or were processed after the initial publication was completed. All samples were obtained with the consent from patients and appropriate approval from ethical committees (REC ref 07/H0308/161) at the University of Cambridge and the British Columbia Cancer Research Centre. Only primary tumors and adjacent normal tissues from female patients were retained. Detailed information about tissue collection for each site can be found in the original METABRIC publication^6^.

METABRIC clinical data, gene expression profiles, copy number aberration, and point mutation information were used as previously described^6,7^. Clinical and histopathological data including lymph node status, stage, grade, and tumor size, ER status, PAM50 status, and IntClust status were also obtained from these publications. Follow-up from the original study was updated with the latest available records.

### DNA extraction

Sample processing, DNA extractions, and quality assessment were based on the protocols described in the original METABRIC publication^6^. For UK samples, DNA was extracted from 10 × 30-μm sections from each tumor using the DNeasy Blood & Tissue Kit (Qiagen, UK) on the QIAcube (Qiagen) according to manufacturer’s instructions. For Canadian samples, DNA was extracted from 10 to 20 8-μm sections from each tumor using the MagAttract DNA M48 Kit (Qiagen) on the BioRobot M48 (Qiagen) according to manufacturer’s instructions. DNA was quantified with the Qubit Fluorometer (Thermo Fisher Scientific, MA, USA) and quality assessed by gel electrophoresis.

### RRBS library preparation and sequencing

DNA was quantified using Qubit HSdsDNA assay (Life Technologies, CA) and libraries were prepared from a total of 50–100 ng of genomic DNA. A gel-free multiplexed RRBS method was used^28^ with modifications. Briefly, DNA was digested with the restriction endonuclease *MspI*, followed by end repair and A-tailing of the fragments. This is followed by ligation with barcoded methylated TruSeq LT adapters (Illumina). Individual libraries were then quantified using qPCR (KAPA low ROX Library Quantification Kit (KAPA Biosystems) and pooled in 12-plex in equimolar ratios, to allow library balancing. Subsequently, the DNA was bisulfite converted using the Zymo Methylation Gold kit (Zymo Research), as recommended by the manufacturer. RRBS libraries were then PCR-amplified using Pfu Cx DNA Polymerase (Agilent Technologies). Finally, a purification step is conducted to size-select for fragments between 200 and 700 base pairs (bp). The quality of the libraries was assessed using Bioanalyser (Agilent Technologies, CA) and quantified using KAPA low ROX Library Quantification Kits (Kapa Biosystems, MA). A list of all primers used in the RRBS library preparation is included in Supplementary Data 10.

Sequencing was performed on the Illumina HiSeq 2500 (v4 chemistry), with single-end reads of 125 bp length. Multiplexing was conducted at the level of 8 samples per lane. Sequencing was performed by the CRUK CI Genomics Core and de-multiplexing by the CRUK CI Bioinformatics Core.

### Alignment and methylation calling

Trimming of the 3′ ends was performed using Trim Galore! (version 0.3.7: powered by cutadapt) to (i) remove bases with Phred-scaled quality score <20, (ii) remove adaptor contamination, and (iii) remove the additional unmethylated Cs introduced during the end repair step. Reads were aligned to the Human Genome Assembly GRCh37 (UCSC release hg19) performed using *Bismark (version 0.13.1)*^29^. Further processing and CpG extraction was performed using the in-house gpatterns package (version 0.2, https://github.com/tanaylab/gpatterns). Reads with mapping quality (MAPQ) below 30 or reads that had more than 3 non-converted C’s in non-CpG content (CHH) were discarded. Individual CpG methylation was then called for each read, discarding bases with base quality <20.

### Quality assessment

Only samples with more than 1.5 million unique CpGs at a minimum 5× coverage and with a bisulfite conversion level between 99.4 and 99.8% were retained. The identities of those samples with copy number array data available were confirmed by analyzing the samples’ genotypes at loci covered by the AffymetrixSNP6 array. Genotype calls from the sequencing data were compared with those from the SNP6 data that was generated for the original studies. This was to identify possible contamination and sample mix-ups, as this would affect associations with other data sets and clinical parameters.

### Defining promoter and non-promoter methylation

Promoters were defined as 500 bp upstream and 50 bp downstream from a RefSeq TSS (release 69, hg19). In Fig. 1d, active promoters were defined as promoters that had log expression >7 in at least one of the METABRIC samples used in this paper. When analyzing promoter methylation, we use average methylation of all CpGs covered in the promoter region. When analyzing non-promoter methylation, we average all CpGs on one Msp1 fragment. Msp1 fragments that had partial overlap to a defined promoter region or to an exon were excluded.

For annotation of non-promoter elements, we used *HMEC Broad* (GSM733705) and Roadmap *Breast_Luminal_Epithelial* (GSM669595), and *Breast_Myoepithelial* (GSM613870) as downloaded from the ENCODE and Roadmap browser. Putative enhancers were defined as genomic intervals that were at the top 3% of the chip-seq coverage distribution using encode and Roadmap H3K4me1 data, excluding intervals that were within 2 Kbp from annotated promoters. Enhancer intervals were homogenized to 200 bp length by centering and extending to 100 bp on each side.

We used Encode Repliseq data of MCF7 cells (GSM923442) for time-of-replication (TOR) analysis. Loci with values below the median were considered “late” and values that were above the median were considered “early”. In Extended Data Fig. 5b, we divide the Repliseq data into three categories—“late”, “intermediate”, and “early”, that are within the bottom 20%, 20–60%, and >60% percentiles, respectively.

### Epi-polymorphism quantification

For pattern distribution analysis, we have used the approach described in refs.^20,22^. To reanalyze the bisulfite sequencing data at the read-level we grouped RRBS reads by their mapped *Msp1* restriction fragment and identified the 5 most covered CpG loci within each fragment. If <5 CpGs were present on the restriction fragment, we have filtered it from further epi-polymorphism analysis. We then projected all RRBS reads on the high-frequency CpGs to create homogenized methylation reads (k-mers) of length 5 for each *Msp1* fragment, further filtering reads that fail to cover all selected CpGs. Then, we down-sampled the retained homogenized reads of each restriction fragment to generate a set of exactly 30 reads for each locus in each tumor, or defined the relevant fragment as uncovered in case <30 homogenized reads were available. We then compute epi-polymorphism as described in ref. ^20^. When stratifying epi-polymorphism given average methylation, we always recomputed the averages from the homogenized, subsampled dataset.

### TME normalization strategy

To facilitate robust deconvolution of these tumor microenvironment (TME) effects, *Methylayer* uses an unsupervised approach relying on analysis of the cross-correlations between gene expression profiles with promoter methylation signatures.

In broad strokes, *Methylayer*’s normalization strategy is to (A) Compute cross-correlation between gene expression and promoter methylation. (B) Cluster the cross-correlation matrix to identify TME expression signatures (i.e., groups of TME genes that affect promoter methylation). (C) Use the Euclidean distance in the 2D space of these signatures to identify the K-nearest neighbors of each tumor. (D) Subtract from the raw-methylation value of each tumor the mean methylation of its K neighbors.

#### (A, B) Basic cross-correlation of expression and methylation profiles

We matched promoter methylation and gene expression profiles using Refseq annotations. Alternative promoters were resolved by selecting the promoter with the minimal average methylation value in the normal samples.

Cross-correlation matrices were generated (Extended Data Fig. 2a, b) by computing Pearson correlation between log-transformed expression levels and raw-methylation levels of promoters. We computed these values separately for ER+/ER− and normal samples. Loci with mean methylation value lower than 0.1 or higher than 0.9 were excluded. We used only rows (expression profiles) and columns (methylation profiles) that had at least one correlation value greater than 0.25 or smaller than −0.25. This gave rise to a matrix on 2701 loci and 5879 genes (3525 and 11,054 in ER−) that were clustered using hierarchical clustering on the Euclidian distances, with agglomeration method “ward.D2”. Thirty clusters were then extracted by cutting the tree.

#### (C, D) TME normalization

We used the ER+ expression–methylation cross-correlation clusters to identify an “Immune” gene cluster (clusters CE2, including ‘*CD3D*’ and additional 195 genes) and a “CAF” gene cluster (CE8, including ‘*CAV1*’ and additional 207 genes). We then computed the immune and CAF signatures of the ER+ tumors using the mean expression of the two clusters. Using Euclidean distance in the 2D space of these signatures, we identified the K-nearest neighbors (using K = 30) of each tumor. For normalization, we subtracted from the raw-methylation value of each tumor the mean methylation of its 30 neighbors. For example, gene expression associations of *CAV1*, the canonical CAF gene, and *CD3D*, the canonical T-cell gene have been normalized while cancer-relevant genes such as *GATA3* and *TOP2A* were not affected by our normalization (Fig. 1h). We studied the impact of increasing or decreasing the total number of neighbors (Extended Data Fig. 3e). It should be noted that using smaller K values will increase noise (since the neighborhood mean methylation will become less stable), while using larger K values may lead to less effective normalization of the CAF and immune signatures (since the neighborhood becomes less homogeneous in the Immune/ CAF space).

For normalizing ER− tumors and normal samples, we used a similar procedure but reduced the K parameter to 15 in order to accommodate smaller number of samples and more homogeneous CAF/immune distribution. For ER− tumors, the immune/ CAF expression clusters were CE16 (with 345 genes) and CE18 (360 genes). For normal samples, these clusters were defined as CE3 (864 genes) and CE11 (592 genes).

We derived the TME methylation scores by averaging the methylation of all promoters that were negatively correlated (<−0.3) with the immune and CAF expression scores.

More details regarding TME normalization can be found in the *Methylayer* R package: https://github.com/tanaylab/methylayer.

### Independently inferred estimates of immune and CAF fractions

The validity of *Methylayer* Immune and CAF expression modules were assessed with deconvoluted gene expression profiles for the METABRIC samples (defined using the MCP-counter with standard parameters, a gene signature-based method as described in ref. ^30^. H&E Digital pathology data as well as Imaging Mass Cytometry (IMC) based fractions for the METABRIC samples were also available for comparison. An additional, independent and unsupervised analysis scheme using non-negative matrix factorization (NMF) further validated *Methylayer* estimates of Immune and CAF (Supplementary note 1).

### Definition of clock, MG, and ML scores

We sampled 50,000 normalized methylation signatures of ER+ tumors (promoters and non-promoters) with mean methylation <0.1 and removed loci that did not have at least one absolute correlation value >0.25. We then clustered the correlation matrix of these signatures with hierarchical clustering, with agglomeration method “ward.D2” and identified three major clusters (Extended Data Fig. 4a, b). We defined the clock, MG, and ML as the mean normalized methylation of the loci within these clusters.

In summary, five methylation scores were defined. Two of these (Immune, CAF) were based on raw-methylation levels of loci correlated with Immune and CAF gene expression, respectively. The other three were defining large correlation clusters of normalized methylation in ER+ and were projected also to ER− and normal samples. Supplementary Data 5 contains the scores for each ER+ tumors, ER− tumors, and normal METABRIC samples, and Supplementary Data 7 contains the loci from which each score was derived. An additional, independent and unsupervised analysis scheme using non-negative matrix factorization (NMF) further validated all five methylation scores derived using the *Methylayer* pipeline (Supplementary note 1).

### Screening for *cis*-regulated promoters and enhancers

The approach we take for screening for *cis*-regulated promoters involves three stages. First, we compute the expression–methylation correlation for each promoter and each gene. Second, for every gene, rank its correlations with promoters, and look at the rank of the gene’s own promoter. Finally, we estimate the significance of the rank of the gene’s own promoter. If the highest (negative) correlation of a gene is with its own promoter there is a high probability that this correlation is specific (*in cis*) and not a part of a large methylation effect that correlates many loci with multiple genes (*in trans* effect).

Pairing of promoter methylation and gene expression profiles was defined using Refseq coordinates, with ambiguity resolved using analysis of minimal methylation in normal samples. This provided a potential *cis*-correlation value for 9360 genes for which promoter coverage was sufficiently high, and were not part of the immune/CAF expression modules.

Working separately on ER+ and ER− samples, we formed a 9360 × 9360 cross-correlation matrix (Pearson) by matching all expression profiles (columns) to all methylation profiles (rows). Correlations that were based on <50 samples were removed. We then ranked each column (lowest to highest, rank 1 being the strongest negative correlation), and estimated the significance of the rank values on the diagonal (representing the putative *cis*-interactions). Assuming independence between expression and methylation, the false discovery rate (FDR) for detecting the *cis*-target (diagonal value) with rank value <= *k* was defined as *k*/*m*, where *m* is the number of observed genes having *cis*-target (diagonal value) with rank value <= *k*. Supplementary Data 8 contains the candidates when limiting the FDR to at least 0.05 (*k* = 52), 0.01 (*k* = 6) 0.005 (*k* = 2), and also when considering only the promoter with the rank = 1 (FDR < 0.003).

To search for non-promoter *cis*-regulation candidates we used a modified procedure to the promoter screen, circumventing the lack of one-to-one correspondence between loci and regulatory targets. Since a locus can be correlated *in cis* with multiple genes, we rank the correlation of every locus with all the genes, and then examine the ranks of genes that are within 500 kb to the locus.

Specifically, we generated a matrix of correlation between 185,389 non-promoter (at least 2 kb from an annotated promoter) loci and the 15,497 gene expression profiles. For each expression profile, we defined a genomic coordinate based on its Refseq annotation (note that this analysis included also some genes without RRBS promoter coverage). We then computed the genomic distance (in bp) between each locus and the coordinate assigned to its most correlated expression profile (both positive and negative correlation). We defined a locus as paired if the TSS of its top (or *k*-highest) correlated gene expression profile was within 500 kb from it. We repeated this procedure on shuffled data and estimated the probability for observing pairing by chance. The false discovery rate of pairing was then estimated as the ratio between pairing events in real and shuffled data.

### UMAP projection of tumors epigenetic signatures

To apply UMAP projection of epigenetic signatures (Fig. 5) for our samples we first attenuated the effect of the CAF and Immune scores by scaling to 1/20, and then ran the UMAP algorithm using R ‘umap’ package (https://cran.r-project.org/web/packages/umap/index.html, version 0.2.2.0) with default parameters. For Clock, MG and ML we used raw methylation (instead of TME-normalized methylation) in order to be able to show normal samples and tumors in the same plot.

### Associating point mutations and copy numbers with the epigenetic scores

We used data on 171 point mutations on 1659 of our samples as described. To assess statistical association between point mutations and epigenetic scores, we have directly compared the distribution of epigenomic scores in tumors with and without each point mutation using Wilcox test. To visualize linkage between epigenomic scores and the mutations we grouped tumors according to 5 strata based on quantiles and showed the frequency of the mutation within each group.

The same approach was used to associate 102 copy number aberration loci to the epigenomic scores, comparing the distribution of epigenomic scores in tumors that gained at least a copy of an oncogene to tumors that did not, and comparing the distribution in tumors that lost a tumor suppressor to those that did not.

### Genetic intra-tumor heterogeneity

To quantify genetic intra-tumor heterogeneity, we used the previously established mutant-allele tumor heterogeneity (MATH) score^31^, which is a tumor-specific score based on the variation in variant allele frequency (VAF) of all mutations in the tumor. 173 genes were profiled for mutations and used for calculation of the MATH score (obtained from ref. ^7^). Chromosomal instability (CIN score) was defined as the percentage of the genome affected by CNAs (obtained from ref. ^7^).

### Survival and progression analysis

Univariable and multivariable Cox proportional hazards models were used to examine the association between the DNA methylation signatures and survival. Breast cancer-specific survival (BCSS) was used as the endpoint. Patients with deaths due to other or unknown causes were censored at the date of death, and all other patients were censored at the date of the last follow-up. All times were further censored at 15 years. For the multivariable models, we included confounding variables: grade, size, lymph node status, age at diagnosis, and IntClust status.

Multistate models were fitted as described in ref. ^8^. Transitions between diagnosis, loco-regional and distant relapse were considered. Patients were stratified into ER status and effects for age, size, grade, number of lymph nodes, and time since relapse were added to the model with different parameters for ER status and current state. The methylation signatures were allowed to have different parameters according to ER and the current state of the patient, but not to non-malignant deaths.

### Reporting summary

Further information on research design is available in the Nature Research Reporting Summary linked to this article.

## Supplementary information

Supplementary Information

Description of Additional Supplementary Files

Supplementary Data 1-10

Reporting Summary

## Data Availability

All primary RRBS data (fastq files) and raw-methylation calls (hg19 and hg38) are deposited at the European Genome-phenome Archive (EGA) under study accession number: EGAS00001004327. Data can be downloaded upon request to EGA (through the METABRIC Data Access Committee). Processed promoter and genomic DNA methylation values (raw and normalized) for the 1538 tumor samples and 244 adjacent normal tissues are available at https://tanaylab.weizmann.ac.il/metabric_rrbs. The genomic copy number, gene expression, somatic mutation, and molecular-subtype information has been described previously^6,7^ and is available at the European Genome-phenome Archive (EGAS00000000083). The TCGA BRCA methylation 450k dataset is available in the TCGA portal (https://cancergenome.nih.gov/). For annotation of non-promoter elements, we used *HMEC Broad* (GSM733705) and Roadmap *Breast_Luminal_Epithelial* (GSM669595) and *Breast_Myoepithelial* (GSM613870) as downloaded from the ENCODE and Roadmap browser. We used Encode Repliseq data of MCF7 cells (GSM923442) for time-of-replication (TOR) analysis. All data described within the Article are available in the Supplementary Data files and at https://tanaylab.weizmann.ac.il/metabric_rrbs.

## Code availability

All analysis code is available for academic use at https://github.com/tanaylab/metabric_rrbs.
