# Supplementary material for: DNA methylation landscapes of 1538 breast cancers reveal a replication-linked clock, epigenomic instability and cis-regulation
Source: Nat Commun. 2021 Sep 13;12:5406. doi: 10.1038/s41467-021-25661-w (PMC8437946; doi:10.1038/s41467-021-25661-w)
Supplement: Supplementary file 1 — Supplementary Information [file 41467_2021_25661_MOESM1_ESM.pdf]

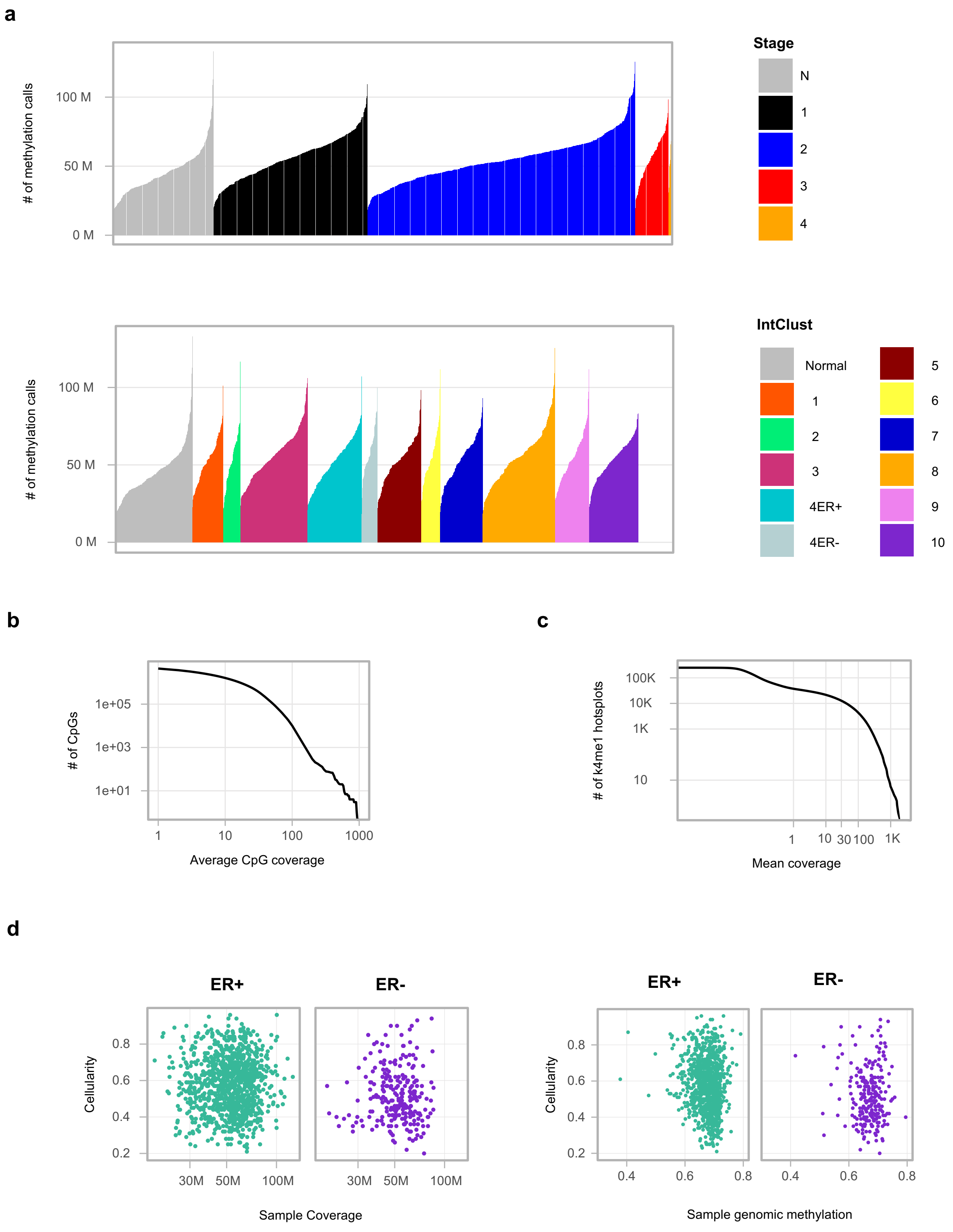

Extended Data Figure 1: METABRIC methylation cohort

a. Total number of methylation calls per METABRIC sample, colored by Stage (top) and Integrative cluster (bottom).
b. Cumulative distribution of mean CpG coverage on the pool of all tumor samples.
c. Mean coverage of H3K4me1 hotspots (CDF) on the pool of all tumor samples.
d. Sample cellularity estimate (based on ASCAT, y-axis) compared to sample coverage (total number of methylation calls, left) and non-promoter methylation (right), in ER+ (green) and ER- (purple) tumors.

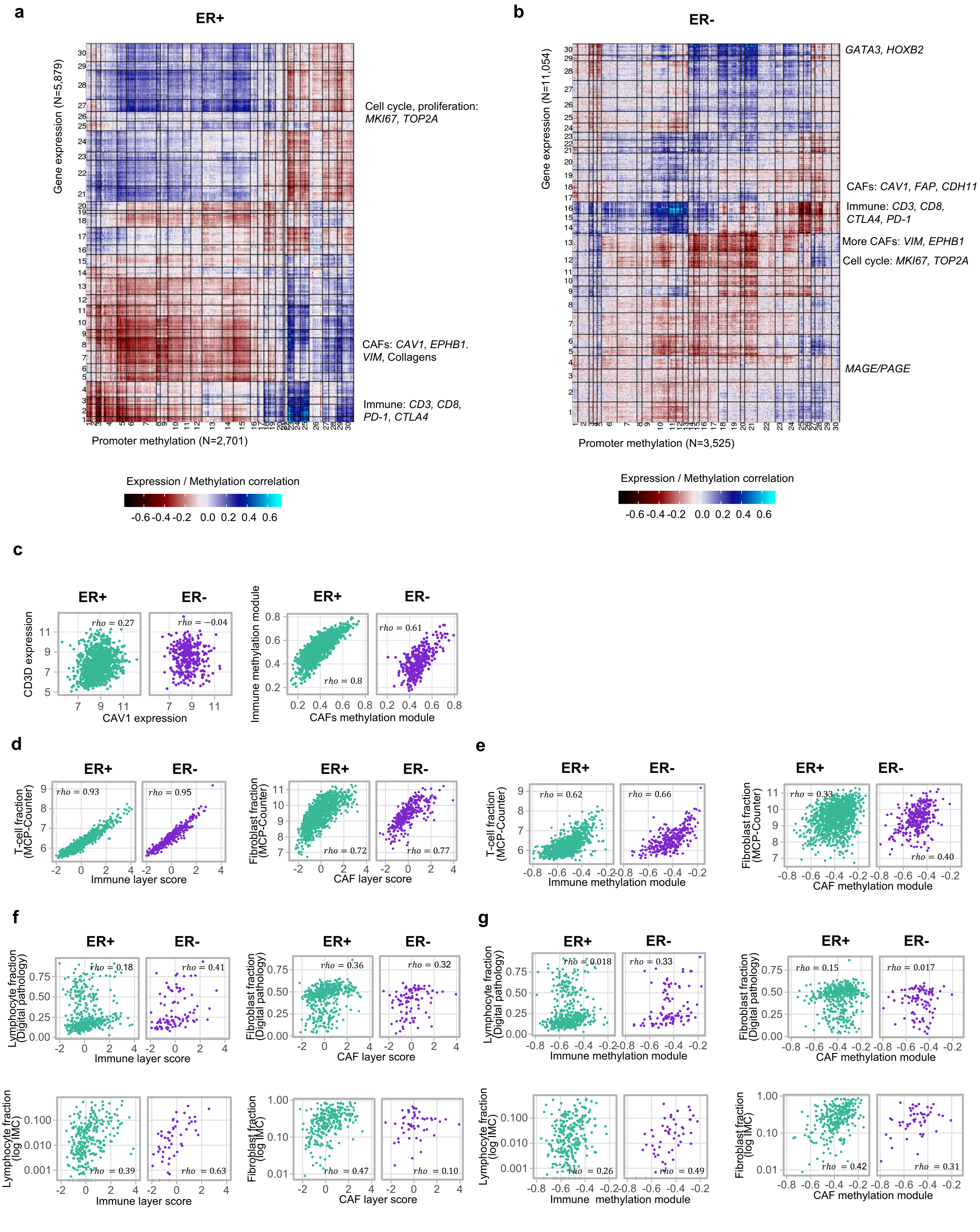

Extended Data Figure 2: The tumor-microenvironment (TME) methylation signatures

Extended Data Figure 2: The tumor-microenvironment (TME) methylation signatures (CONTINUED)

a. Clustered correlation heatmap of tumor gene expression and promoter methylation vectors in ER+ tumors. Expression clusters (CE1-30, rows) are annotated based on key marker gene expression. See **Supplementary Tables 2, 3** for complete details. CAF: cancer associated fibroblasts.

b. Clustered correlation heatmap between methylation profiles (columns) and matching gene expression (rows) in ER- tumors.

c. Comparison of CD3D/CAV1 expression and the Immune/CAF methylation modules.

d. Comparison of *Methylayer* Immune and CAF expression modules (X axis) to independently inferred deconvoluted gene expression profiles (defined using the MCP-counter, Y axis).

e. Comparison of *Methylayer* Immune and CAF methylation modules (X axis) to independently inferred deconvoluted gene expression profiles (defined using the MCP-counter, Y axis).

f. Immune/CAF expression score (x-axis) versus lymphocyte/fibroblasts H&E digital pathology estimates (top) and Imaging Mass Cytometry (IMC, logged) (bottom).

g. Immune/CAF methylation score (x-axis) versus lymphocyte/fibroblasts H&E digital pathology estimates (top) and Imaging Mass Cytometry (IMC, logged) (bottom).

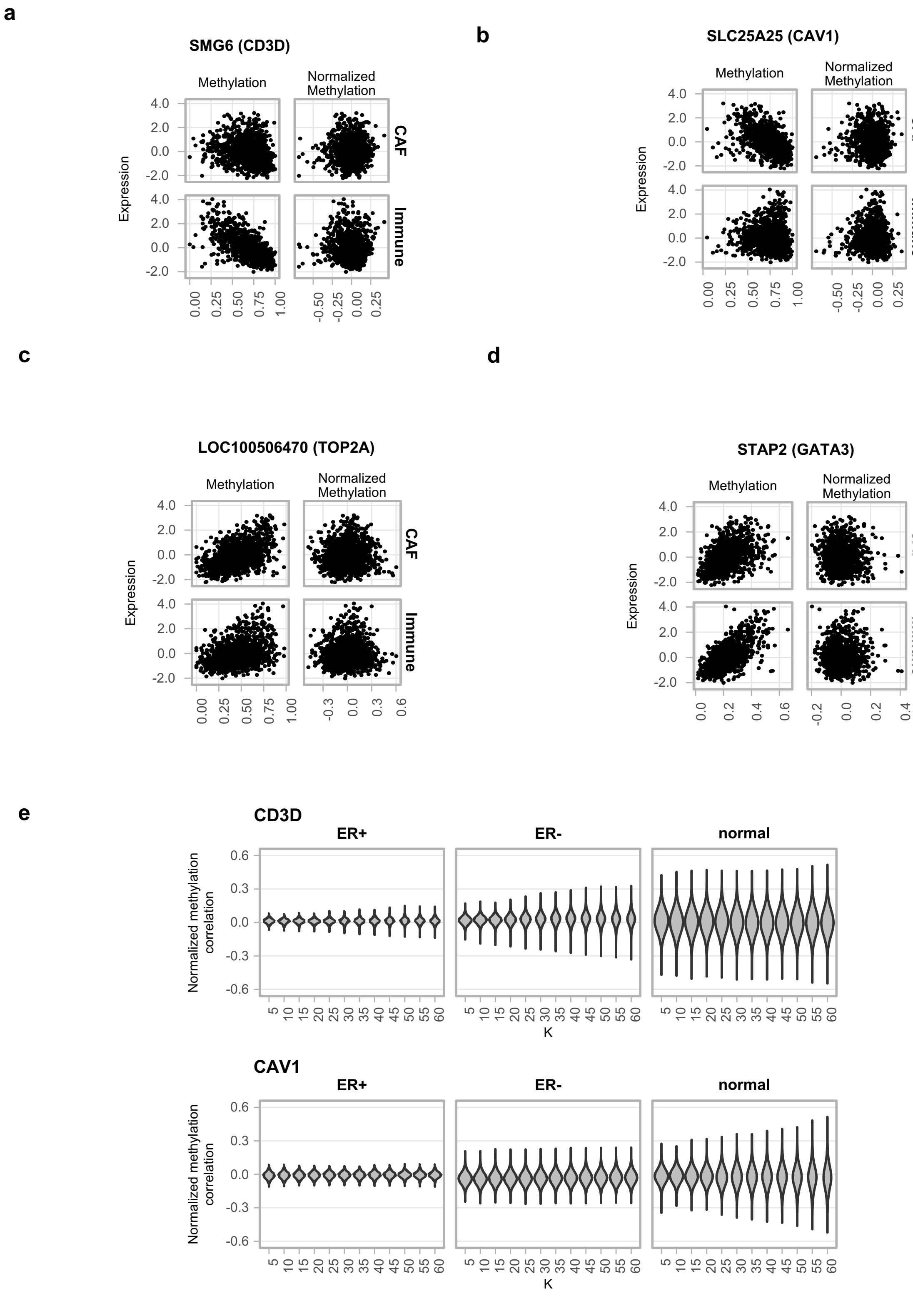

Extended Data Figure 3: Normalizing CAF/Immune methylation

a. CAF (top) and immune (bottom) expression modules for each ER+ sample (y-axis) plotted against methylation of the most correlated promoter to *CD3D* gene expression (SMG6) before (left) and after (right) normalization.

b-d. Same as a. for SKC25A25 (most correlated to *CAV1*), LOC100506470 (most correlated to *TOP2A*) and STAP2 (most correlated to *GATA3*) promoter methylation.

e. Distribution of promoter methylation correlation to the expression of *CD3D* (top) and *CAV1* (bottom) for different K parameters. Larger values of K lead to less effective normalization (wider correlation distribution) of the CAF and immune signatures since the neighborhood becomes less homogenous in the Immune/CAF space.

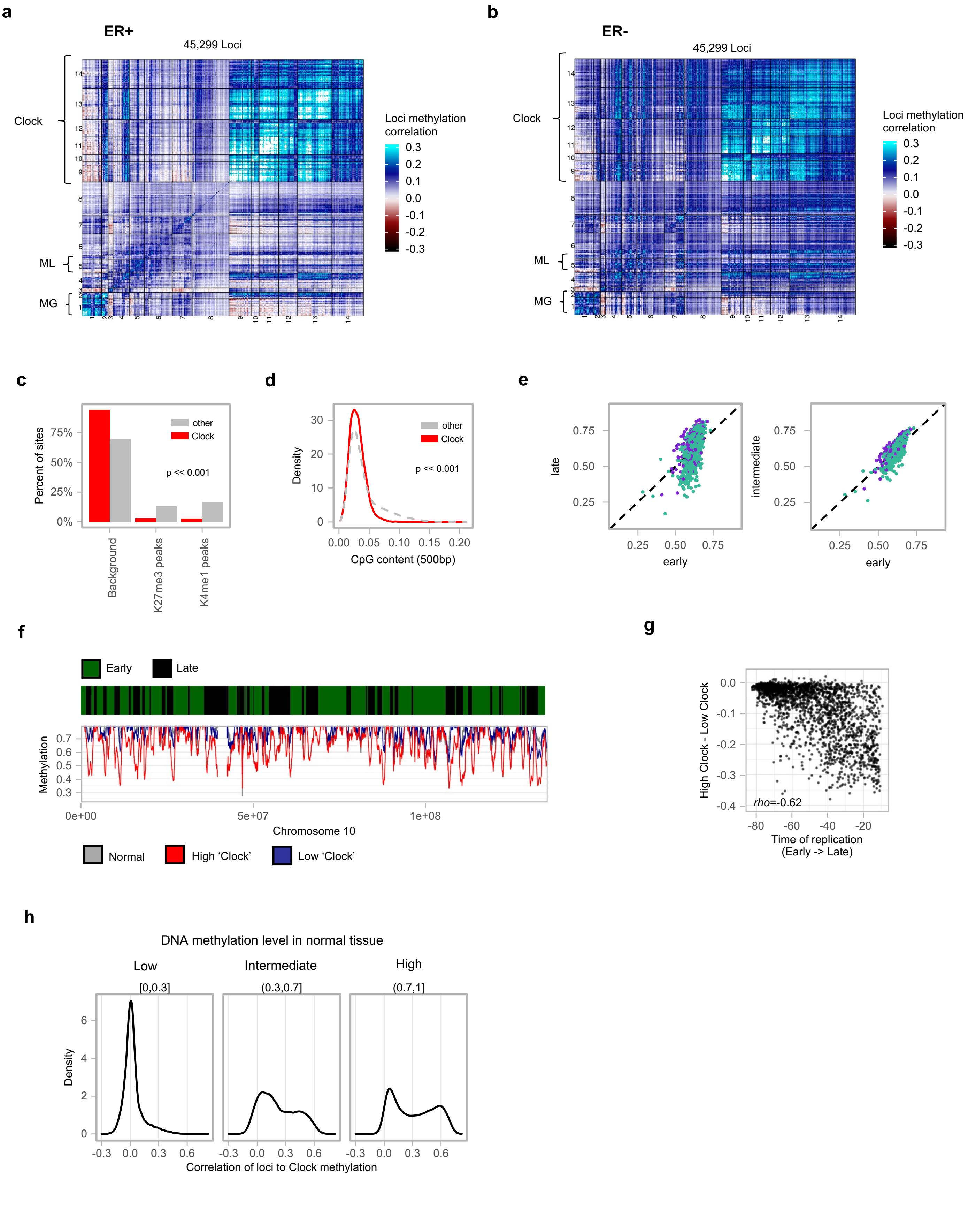

Extended Data Figure 4: Loss clock distributions

Extended Data Figure 4: Loss clock distributions (CONTINUED)

a. Clustered correlation heatmap of normalized methylation profiles for 45,299 loci in ER+ tumors. Detected locus clusters are annotated at the left (numbers), and layers are constructed from such clusters as labeled (Clock, ML, MG).

b. Similar to a, for ER- samples, where loci order is determined by the ER+ clustering.

c. Distribution of epigenomic context for loss clock versus other methylation loci. (χ^2 test, $p < 2.2e-16$).

d. Distribution of CpG content for loss clock versus other loci. (two-tailed Kolmogorov-Smirnoff test, $p < 2.2e-16$).

e. Average methylation in early versus late replicating regions (left) and early versus intermediate replicating regions (right) for each tumor sample.

f. Similar to Fig 1m, showing data on chromosome 10.

g. Plotted is the difference in methylation between samples with high and low clock score in genomic bins of 0.5MB of chromosome 1 versus their time of replication.

h. Distribution of correlation of every locus with the loss clock loci methylation. Loci are separated into those who have low (0-0.3), intermediate (0.3-0.7) and high (0.7-1) methylation levels in normal tissues.

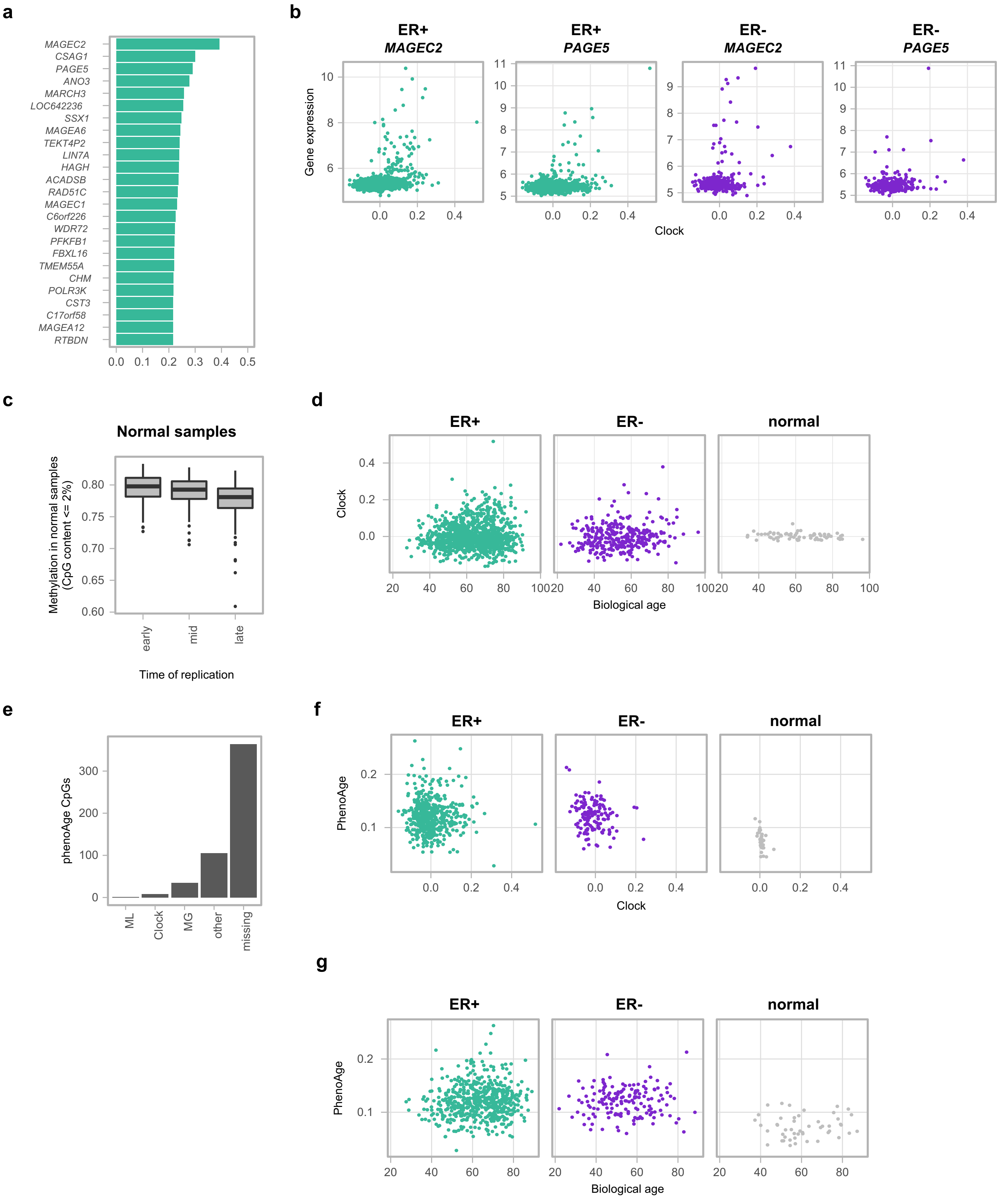

Extended Data Figure 5: Loss clock comparisons

Extended Data Figure 5: Loss clock comparisons (CONTINUED)

a. Gene expression profiles most negatively correlated with the methylation loss clock (in ER+ tumors).
b. Examples (*MAGE2*, *PAGE5*) of correlation between gene expression and loss clock methylation. ER+ (left) and ER- (right) cancers
c. Methylation of normal samples (n = 244) in early, intermediate (mid) and late replication regions. Shown is methylation of CpGs that are of low CpG content (≤ 2% CpGs in the adjacent 500bp, n = 36420). The middle line indicates the median, box limits represent quartiles, and whiskers are 1.5× the interquartile range.
d. Comparison of loss clock layer and sample biological age on tumor and normal samples.
e. Number of CpGs within each of *Methylayer* signatures that are part of the methylation age phenoAge score. Note that coverage for many phenoAge CpGs is not available in RRBS data.
f. Comparing loss clock and estimated phenoAge on tumor and normal samples.
g. Comparing phenoAge and biological age on tumor and normal samples.

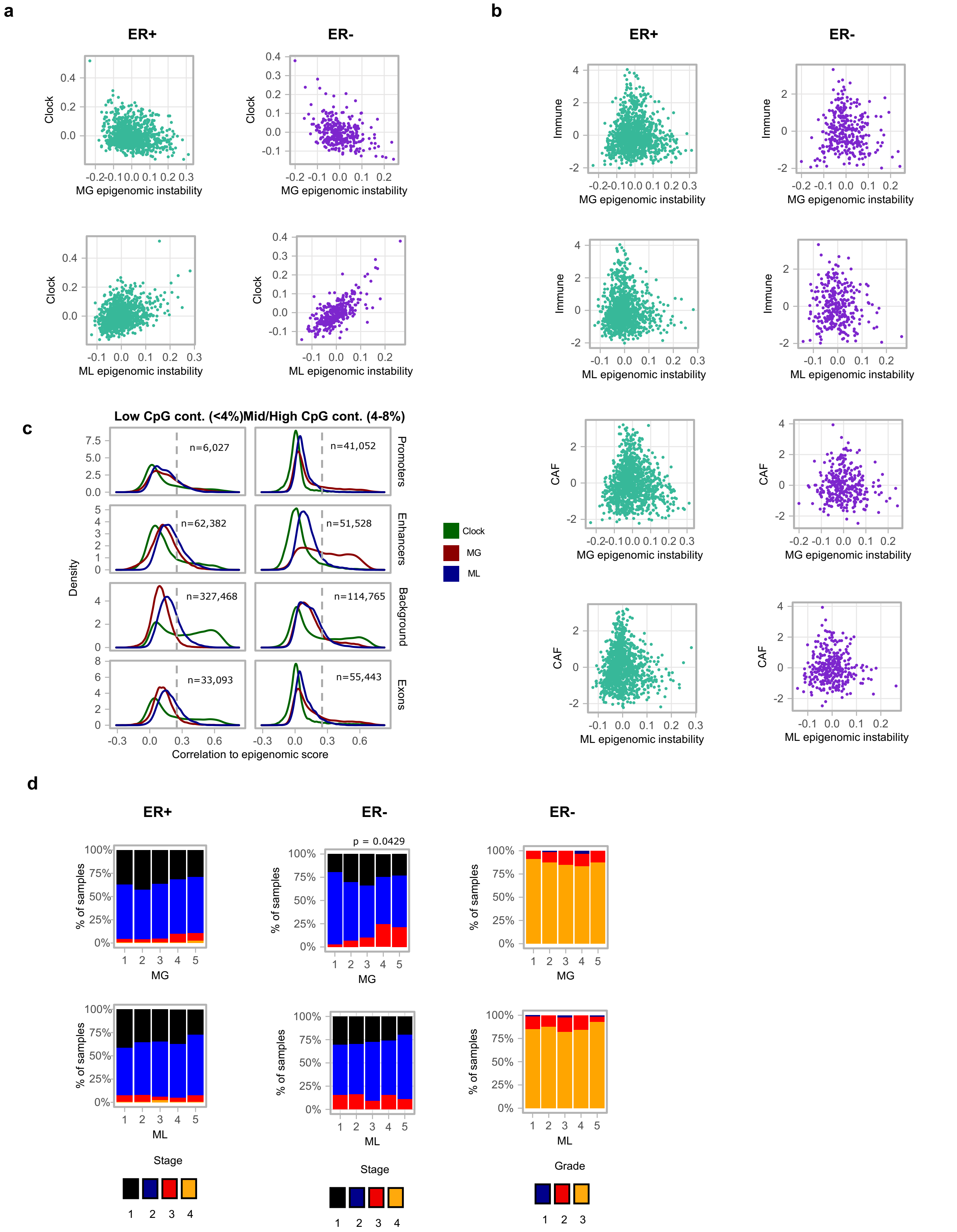

Extended Data Figure 6: Promoter epigenomic instability in breast cancers

Extended Data Figure 6: Promoter epigenomic instability in breast cancers (CONTINUED)
a-b. Comparison of *Methylayer* scores over ER+ and ER- samples separately.
c. Distribution of the correlation between normalized methylation and layer scores (color coded lines) for loci grouped by genomic context and CpG content. Background represents all loci that are not otherwise classified.
d. Distribution of tumor stage and grade stratified by five bins of MG and ML scores. (χ^2 test, tests with $p < 0.05$ are indicated).

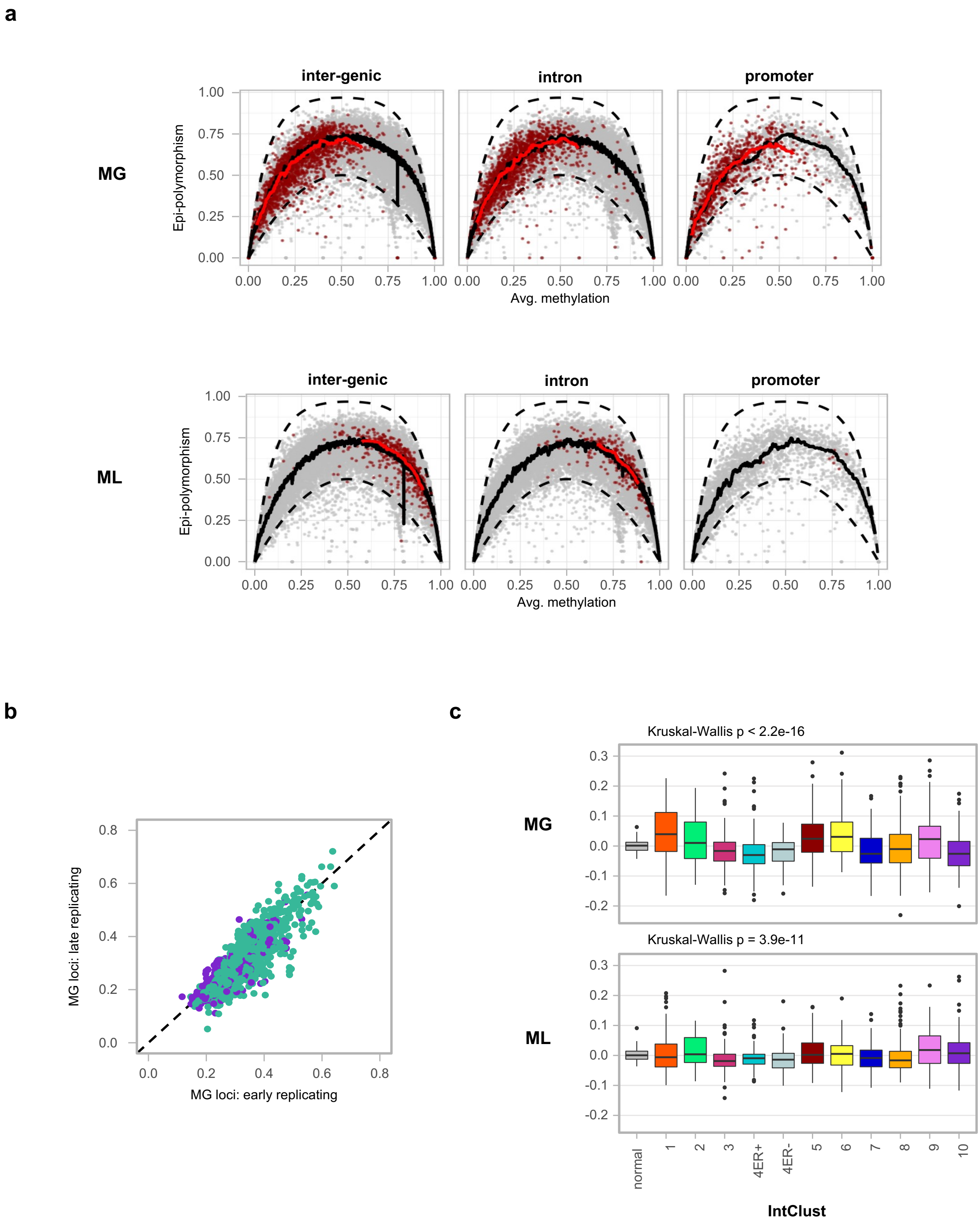

Extended Data Figure 7: Non-promoter epigenomic instability in breast cancers

a. Epi-polymorphism versus average methylation is shown for loci classified by genomic context. Red dots indicate loci correlated with the MG and ML layer scores. Running medians are depicted as black and red lines.

b. Methylation in loci that are part of MG epigenomic instability score in early versus late replication regions. High correlation suggests that MG is not affected by time of replication. Each dot represents a tumor sample, color coded by ER status.

c. Distribution of MG and ML epigenomic instability scores in 1418 breast cancers stratified by Integrative clusters; and in 92 normal breast samples (Kruskal-Wallis test). The middle line indicates the median, box limits represent quartiles, and whiskers are 1.5× the interquartile range.

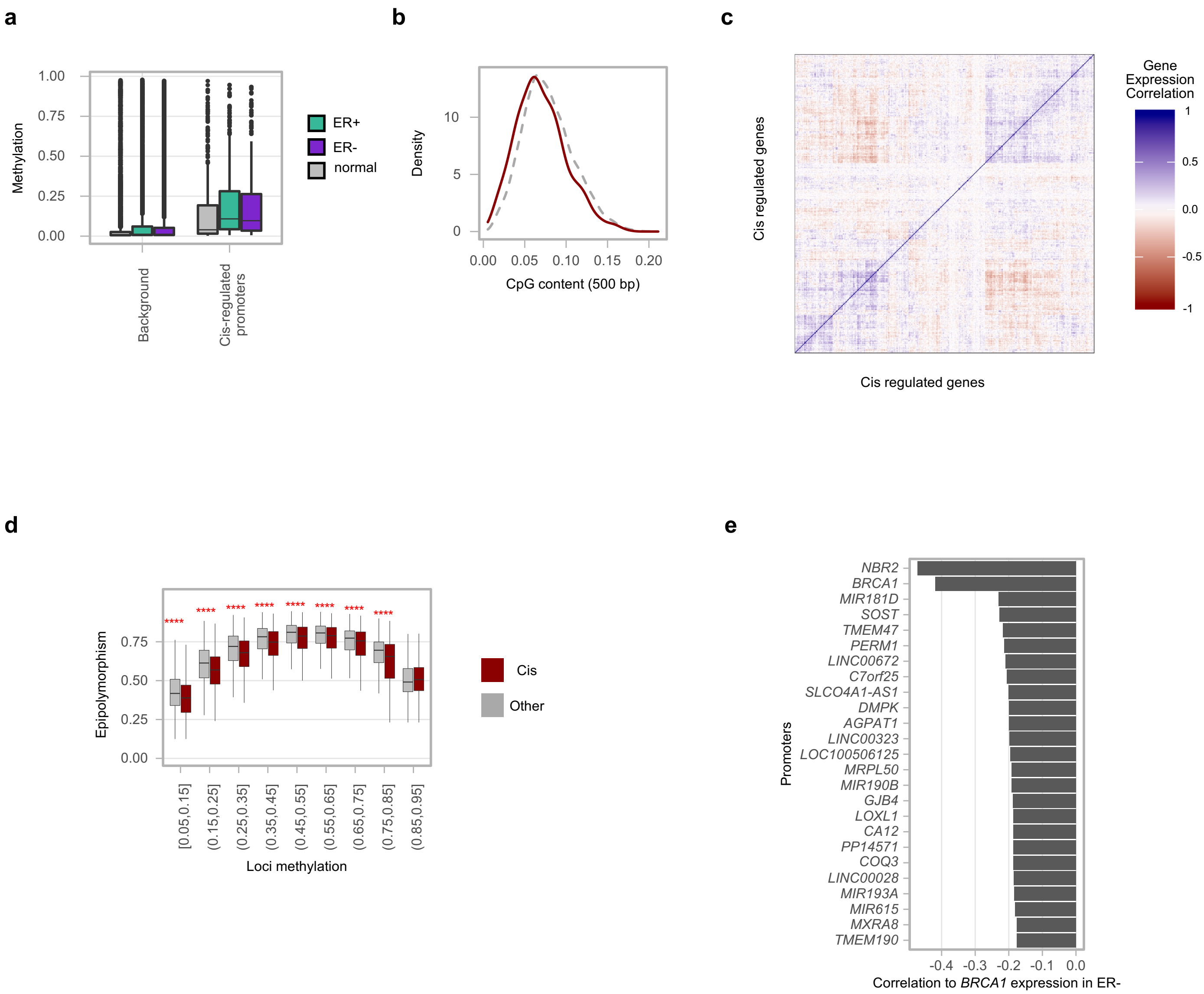

Extended Data Figure 8: Cis-regulation of gene expression

a. Distribution of average methylation in cis-regulated promoters (n = 503) versus other promoters (background, n = 8857).
b. Distribution of CpG content of cis-regulated promoters (red line) and other promoters (dashed gray line).
c. Heatmap of expression correlation between cis-regulated genes.
d. Distribution of Epi-polymorphism in non-promoter loci with high cis E-M correlation. Shown are loci that had at least one tumor sample with average methylation above 0.05 (red, n = 1500). Loci are grouped by average promoter methylation and other loci (gray, n = 88736) are provided for control. *: p ≤ 0.05, **: p ≤ 0.01, ***: p ≤ 0.001, ****: p ≤ 0.0001 (one-sided Wilcox test). The middle line indicates the median, box limits represent quartiles, and whiskers are 1.5× the interquartile range.
e. Top 25 promoters negatively correlating with *BRCA1* expression in ER- tumors.
In all box plots in the figure, the middle line indicates the median, box limits represent quartiles, and whiskers are 1.5× the interquartile range.

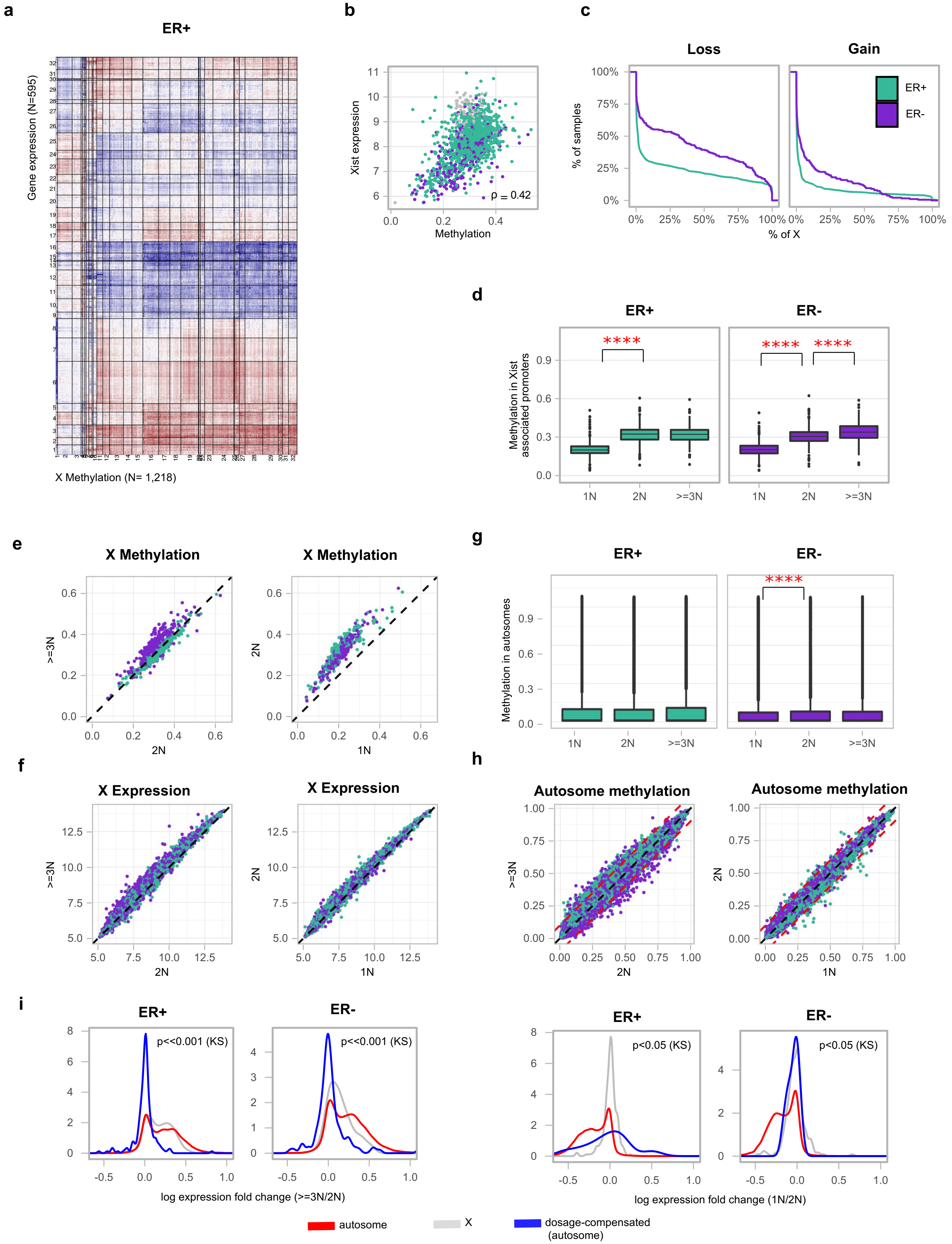

Extended Data Figure 9: X dosage compensation

Extended Data Figure 9: X dosage compensation (CONTINUED)

a. Correlation between 1218 ER+ methylation profiles on the X chromosome (columns) and gene expression (rows).

b. Average methylation of X-linked promoters (correlation with XIST expression ≥ 0.2, n = 615) and XIST expression. Tumor samples color coded by ER status.

c. Cumulative distribution of the percentage of chromosome X that was lost (left) or gained (right) per tumor sample.

d. Methylation distribution of XIST associated promoters in loci that had a single copy (1N) two copies (2N) and 3 and more copies (≥3N) for ER+ (left, n = 444 for 1N, 988 for 2N and 413 for ≥3N) and ER- (right, n = 181 for 1N, 269 for 2N and 135 for ≥3N) tumors. ns: p > 0.05, *: p ≤ 0.05, **: p ≤ 0.01, ***: p ≤ 0.001, ****: p ≤ 0.0001 (spearman *rho*: 2N versus 1N in ER+, p < 2e-16 ; ≥3N versus 2N in ER+, p = 0.86 ; 2N versus 1N in ER-, p < 2e-16 ; ≥3N versus 2N in ER-, p = 7.7e-10).

e. Left: methylation of loci on X chromosome in samples in which they had two copies (x axis) and 3 or more copies (y-axis), colored by ER status. Right: same for samples that lost a copy (x-axis) versus samples with normal X karyotype (y-axis). Lower (higher) correlation suggests higher (lower) dosage compensation.

f. Left: expression of genes on X chromosome in samples in which its promoter had two copies (x axis) and 3 or more copies (y-axis), colored by ER status. Right: same for samples that lost a copy (x-axis) versus samples with normal X karyotype (y-axis). Lower (higher) correlation suggests higher (lower) dosage compensation.

g. Methylation distribution of autosomes in 15207 loci that had a single copy (1N) two copies (2N) and 3 and more copies (≥3N) for ER+ (left, n = 996 for 1N, 1018 for 2N and 1010 for ≥3N) and ER- (right, n = 259 for 1N, 273 for 2N and 276 for ≥3N) tumors. (spearman *rho*: ≥3N versus 2N in ER-, p = 2.6e-05).

h. Left: methylation of loci on autosomes in samples in which they had two copies (x axis) and 4 or more copies (y-axis), colored by ER status. Red dashed lines represent a difference of ±0.1 in average methylation. Right: same for samples that lost a copy (x-axis) versus samples with normal X karyotype (y-axis). Lower (higher) correlation suggests higher (lower) dosage compensation.

i. Left: distribution of log fold change between expression in samples with 2 copies versus 3 or more in ER+ (left) and ER- (right) samples. Red line represents the distribution for loci that are on autosomes. Blue line represents the distribution for loci that had a higher methylation on samples that gained copies (≥3N, above the upper red line in **h** left panel). Gray line represents distribution for loci that are on the X chromosome. Right: distribution of log fold change between expression in samples with 1 copy versus 2 copies. Blue line shows the distribution of loci that had a lower methylation on the samples that lost a copy (1N, below the bottom red line in **h** right panel). *: p ≤ 0.05, **: p ≤ 0.01, ***: p ≤ 0.001, ****: p ≤ 0.0001 (two-tailed Kolmogorov–Smirnov test: 2N versus 1N in ER+, p = 0.0301 ; ≥3N versus 2N in ER+, p < 2e-16 ; 2N versus 1N in ER-, p = 0.0371 ; ≥3N versus 2N in ER-, p < 2e-16).

In all box plots in the figure, the middle line indicates the median, box limits represent quartiles, and whiskers are 1.5× the interquartile range.

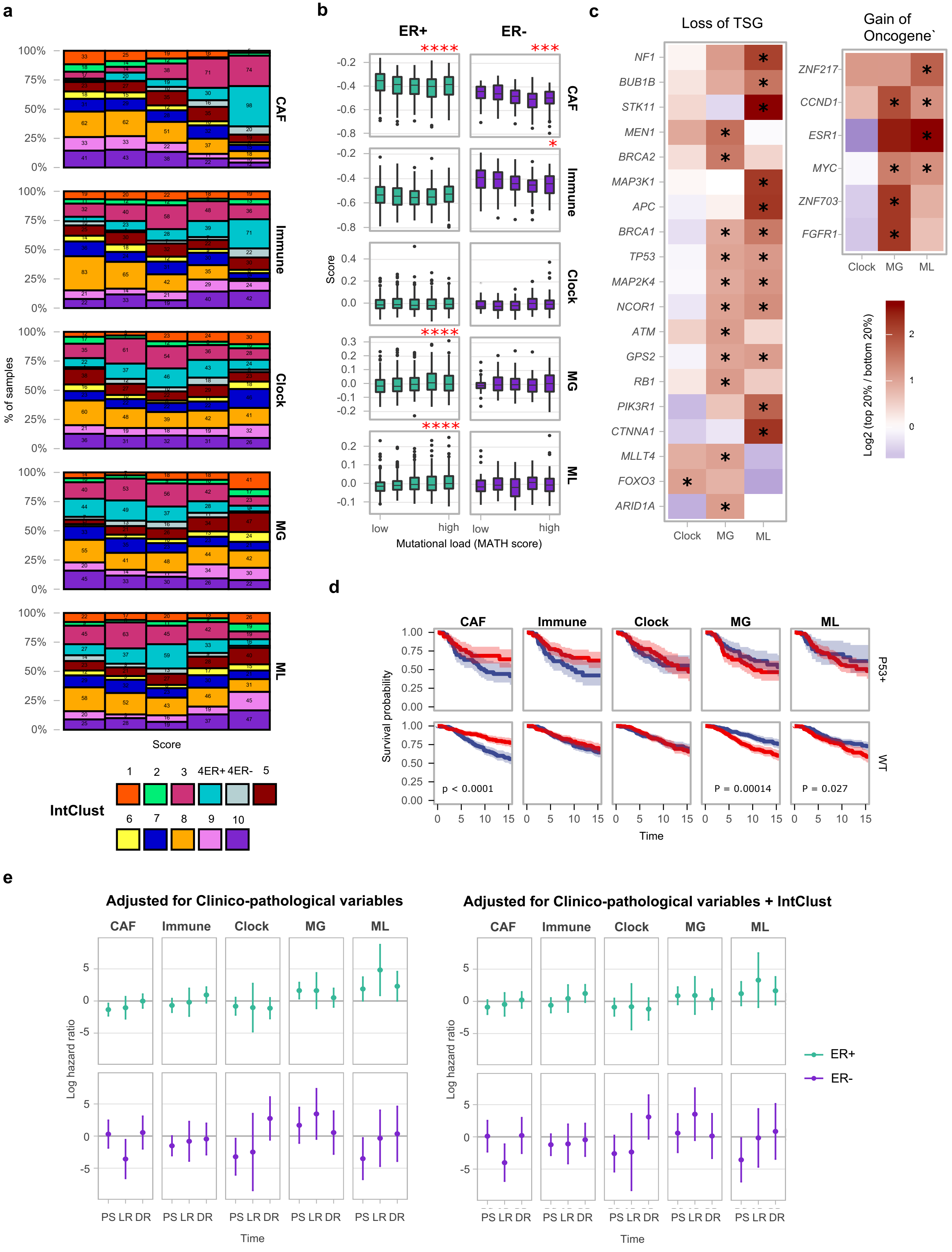

Extended Data Figure 10: Epigenetic scores in the genomic and clinical context of breast cancer

Extended Data Figure 10: Epigenetic scores in the genomic and clinical context of breast cancer (CONTINUED)
a. Epigenomic scores across the 11 integrative clusters (IntClust). Each score was stratified into 5 bins (bars), and shown are the proportions of each integrative cluster in each bin.
b. Boxplots of distribution of epigenomic signatures in 1024 ER+ and 263 ER- tumors stratified according to their genomic intra-tumor heterogeneity score (using mutant-allele tumor heterogeneity (MATH) score). *: $p \leq 0.05$, **: $p \leq 0.01$, ***: $p \leq 0.001$, ****: $p \leq 0.0001$ (spearman *rho*). The middle line indicates the median, box limits represent quartiles, and whiskers are 1.5× the interquartile range.
c. Left: Plot of log2 fold change between epigenetic scores in the top and bottom 20% of tumors that had loss of heterozygosity (LOH) in one of 57 tumor suppressor genes in ER+ tumors. CNAs with statistically significant association to an epigenomic score ($p<0.01$, two-sided Wilcox-test, FDR corrected over 102 tumor associated genes) are highlighted with a star. Shown are only genes that were associated with at least one score.
Right: Same for gain of copy in one of 28 oncogenes in ER+ tumors.
d. Kaplan-Meier survival plots for ER+ tumors with P53+ mutation (top, n = 202) and without P53+ mutation (bottom, n = 824), grouped into high-scoring and low-scoring groups for each epigenomic signature. 95% confidence intervals are shown. Log-rank p-values for survival difference are reported .
e. Multi-state breast cancer progression models[8]. Log Hazard Ratios (mean with 95% confidence intervals) calculated for each epigenomic signature at different states (post-surgery (PS), after locoregional relapse (LR) and after distant relapse (DR)). Models were stratified for ER+ (top, n = 1079) and ER- (bottom, n = 306) status. Left: CP (adjusted for Clinico-pathological variables - age, grade, tumor size and lymph node status). Right: CP + IntClust (adjusted for Clinico-pathological variables and Integrative cluster subtypes).

SUPPLEMENTARY NOTE 1

Validation of *Methylayer* pipeline using an unsupervised approach based on non-negative matrix factorization

In order to validate the (CAF, Immune), MG, ML and Clock signatures identified using the *Methylayer* pipeline, an unsupervised computational framework was utilized to conduct non-negative matrix factorization (NMF) of the METABRIC methylomes (1538 breast tumor and 244 normal breast samples) into latent methylation components (LMCs). In order to strengthen the accuracy of recovering normal cell-type components from the heterogeneous breast methylomes, 10 normal reference methylomes (WGBS) were included in the analysis - Roadmap breast luminal epithelial cells; Roadmap breast myoepithelial cells; 2 x Roadmap fibroblast primary cell lines; 2 x Blueprint adult endothelial progenitor cells (S00UJK AND S01GU9); 4 x Blueprint CD8+ alpha beta T-cells (C00256, C003VO, C0066P and S00C2F). Specifically, the reference methylomes were first filtered for those that overlapped with the METABRIC RRBS data (n=93,703 CpG sites). Missing methylation values were imputed using mean-imputation with a regularization parameter (k=4). CpGs were filtered based on having an SD > 0.2 either among the 1538 breast tumors or among the 10 reference methylome; and then 20,000 CpGs were randomly selected to be used as input in the deconvolution. The MeDeCom algorithm[1] based on NMF was applied on the 1792 samples (1782 METABRIC + 10 reference samples) over the 20,000 CpG sites to deconvolute the methylomes into independent LMCs. Selection of the number of LMCs (k=8) and regularization factor (lambda=0.01) were based on cross-validation.

The 8 LMCs were compared (spearman correlation) with the 10 normal reference methylomes (**Fig 1**). Both the fibroblast cell lines and myoepithelial cells were most associated with LMC1 indicating that LMC1 might represent the stromal fraction of the breast tumors. In addition, LMC1 also showed high correlation with endothelial cells. However, both the endothelial cell lines but particularly, the 4 T-cell lines were most associated with LMC5 suggesting that it represents the immune infiltrating fraction of the breast tumors.

Next, the 5 layers defined using the *Methylayer* pipeline were investigated (**Fig 2**). Each of the 5 layers (caf, immune, clock, MG and ML) were strongly correlated (spearman) with independent LMCs for ER+ and ER- breast tumors. The CAF methylation module was significantly correlated with LMC1 (r=0.82, p<<0.001 in ER+; r=0.68, p<<0.001 in ER-). The immune methylation module was significantly correlated with LMC5 (r=0.78, p<<0.001 in ER+; r=0.79, p<<0.001 in ER-). The Clock signature was significantly correlated with LMC2 (r=0.61, p<<0.001 in ER+; r=0.72, p<<0.001 in ER-) and with LMC3 (r=0.7, p<<0.001 in ER+; r=0.49, p<<0.001 in ER-). The MG epigenetic instability signature was significantly correlated with LMC7 (r=0.78, p<<0.001 in ER+; r=0.82, p<<0.001 in ER-) and the ML epigenetic instability signature was significantly correlated with LMC6 (r=0.51, p<<0.001 in ER+). Two remaining LMCs (LMC4 and LMC8) were characterized by ER+ and ER- specific hypomethylation (recall *Methylayer* was applied on the two sub-cohorts separately).

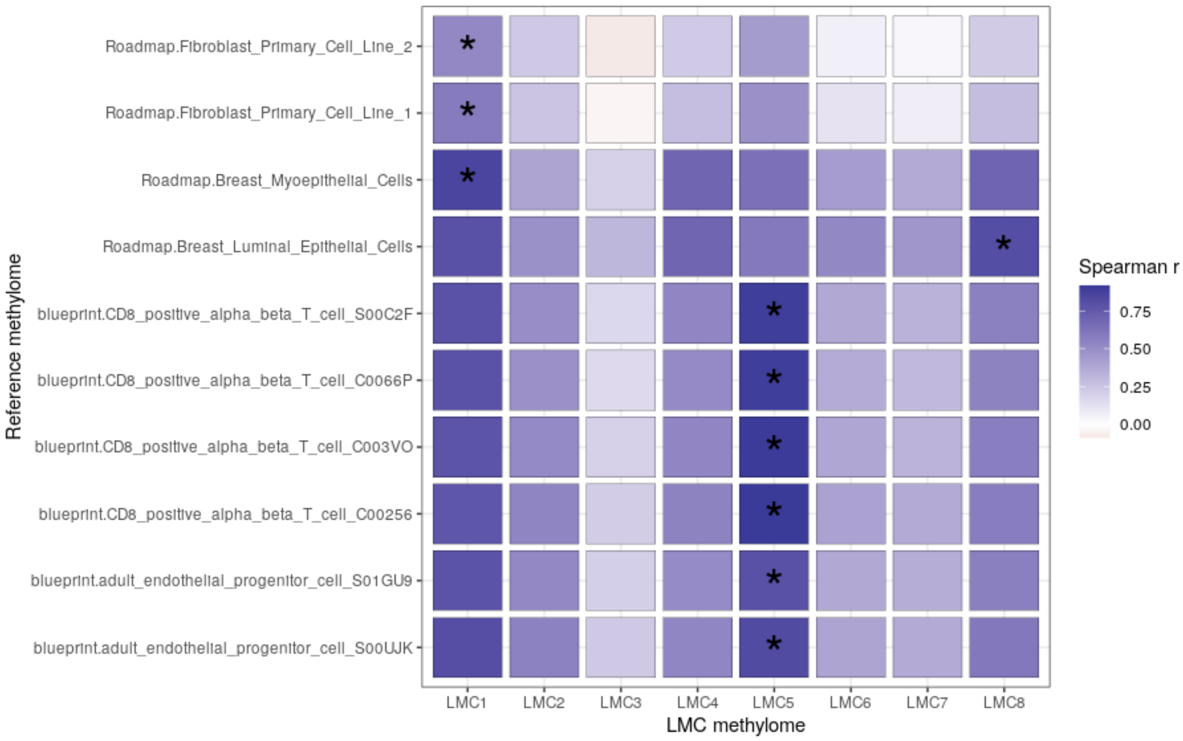

Supplementary Figure 11: Comparison of the 8 LMCs with the 10 normal reference methylomes. Color of the tiles represents spearman correlation. For each reference methylome, the LMC that is most correlated is indicated with an '*'.

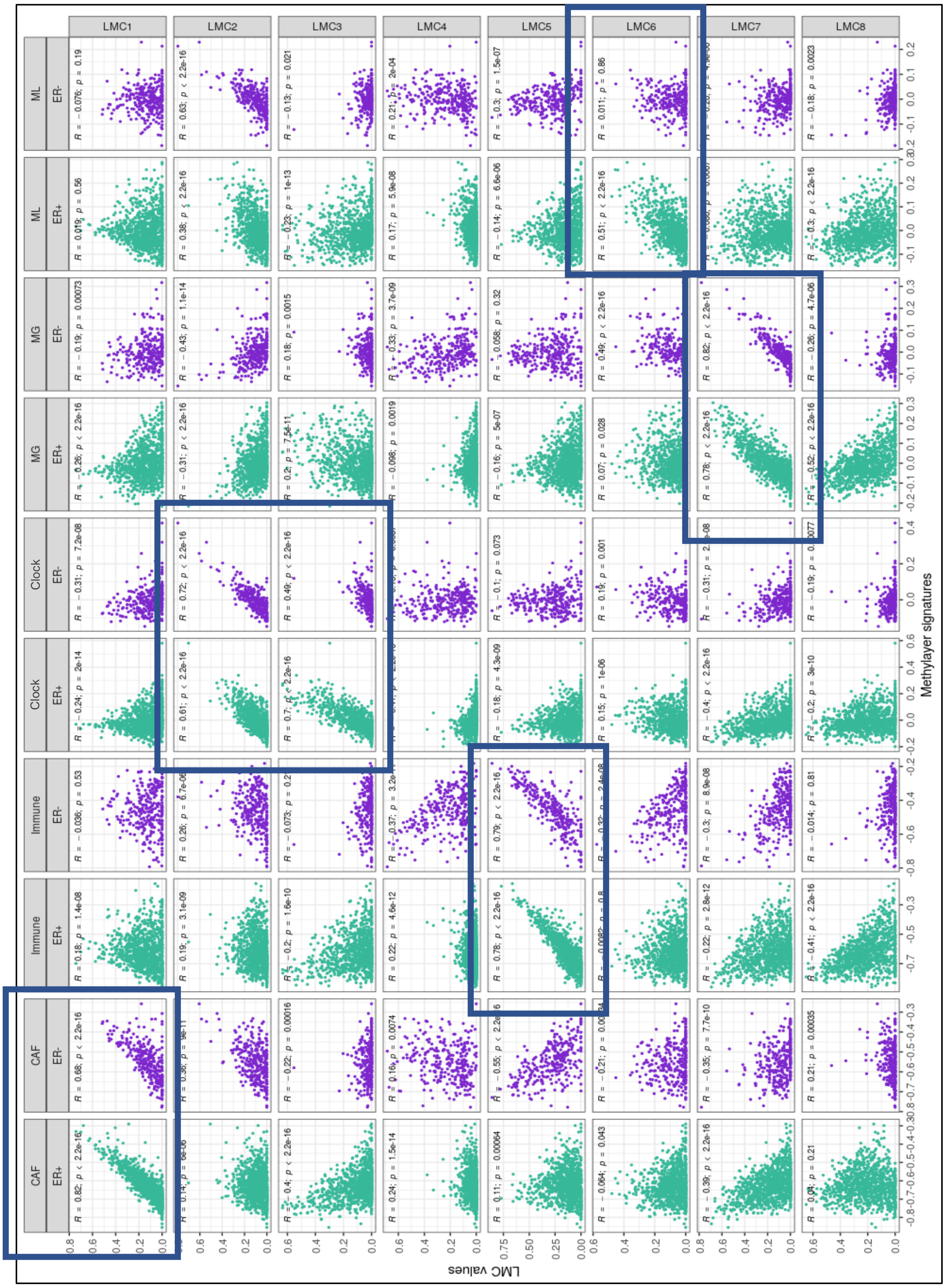

Supplementary Figure 12: Robustness of the *Methylayer* pipeline confirmed by an unsupervised approach based on non-negative matrix factorization (NMF).
Each of the 5 signatures (CAF, immune, Clock, MG and ML) defined using the *Methylayer* pipeline were strongly correlated with independent LMCs derived by NMF for both ER+ and ER- breast tumors.

SUPPLEMENTARY NOTE 2

Applying *Methylayer* to TCGA breast cancer dataset

Table of Contents

Import and load TCGA breast cancer methylation and expression data 5

Normalize tumor microenvironment (TME) effects 7

Normalize ER+/ER-/normal TCGA-BRCA samples 8

Merge all normalized methylation 8

Merge all TME expression scores 8

Diagnose TME normalization 9

Define epigenomic scores 10

Compare TCGA-BRCA epigenomic modules to METABRIC 15

Correlation between the features 18

Methylation regulation of gene expression in cis 22

Load normalized methylation and separate it to promoters and non-promoters 23

Promoters 23

Compare to METABRIC cis regulated genes 25

Non-promoters 26

Appendix 1: additional TME normalization diagnostics 29

Import and load TCGA breast cancer methylation and expression data

We use the following Bioconductor packages to load TCGA-BRCA data:

- *curatedTCGAData*
- *TCGAutils*

We take the **BRCA_Methylation_methyl450-20160128** dataset for 450k array methylation data and **BRCA_RNASeq2GeneNorm-20160128** for expression data. Methylation data is saved as *misha track.array*.

Import is done by running:

```
import_breast_tcga()
```

See *scripts/tcga/import.r* at *tanaylab/metabric_rrbs* for details.

Loading is done by running the following commands:

```
init_tcga_samp_data()
head(tcga_samp_data)

## # A tibble: 6 x 20
##   samp_id barcode submitter_id sample_definiti… sample vial  portion an
alyte
##   <chr>   <chr>   <chr>        <chr>             <int> <chr>   <int> <c
hr>
## 1 TCGA_3… TCGA-3… TCGA-3C-AAAU Primary Solid T…      1 A          11 D
## 2 TCGA_3… TCGA-3… TCGA-3C-AALI Primary Solid T…      1 A          11 D
## 3 TCGA_3… TCGA-3… TCGA-3C-AALJ Primary Solid T…      1 A          31 D
## 4 TCGA_3… TCGA-3… TCGA-3C-AALK Primary Solid T…      1 A          11 D
## 5 TCGA_4… TCGA-4… TCGA-4H-AAAK Primary Solid T…      1 A          12 D
## 6 TCGA_5… TCGA-5… TCGA-5L-AAT0 Primary Solid T…      1 A          12 D
## # … with 12 more variables: plate <chr>, center <int>, type <chr>, ER <
chr>,
## #   HER2 <chr>, PR <chr>, gender <chr>, PAM50 <chr>, age <int>, stage <
chr>,
## #   IHC <chr>, patient <chr>
```

Load TCGA-BRCA expression and methylation data. Methylation data is separated to promoters and non-promoters.

```
load_all_tcga_brca_data()
```

Expression data:

```
dim(tcga_expr)

## [1] 20501  1212

dim(tcga_expr_positive)

## [1] 20501   594

dim(tcga_expr_negative)
```

```
## [1] 20501   179
dim(tcga_expr_normal)
## [1] 20501    89
```

Promoter methylation data:

```
dim(tcga_prom_meth)
## [1] 32378   885
dim(tcga_prom_meth_positive)
## [1] 32378   358
dim(tcga_prom_meth_negative)
## [1] 32378   111
dim(tcga_prom_meth_normal)
## [1] 32378    82
```

Non-promoter methylation data:

```
dim(tcga_genomic_meth)
## [1] 176342    885
dim(tcga_genomic_meth_positive)
## [1] 176342    358
dim(tcga_genomic_meth_negative)
## [1] 176342    111
dim(tcga_genomic_meth_normal)
## [1] 176342     82
```

Combined promoters and non-promoter methylation:

Note: The full matrix for TCGA-BRCA methylation has a row per **CpG** whereas for normalization we averaged the CpGs of every promoter.

```
dim(all_meth)
## [1] 394182    886
dim(all_meth_positive)
## [1] 394182    358
dim(all_meth_negative)
## [1] 394182    111
dim(all_meth_normal)
```

```
## [1] 394182     82
```

Normalize tumor microenvironment (TME) effects

Sampling DNA methylation from bulk tumor tissue is known to be affected by variable populations of stromal and immune cells. Mean methylation levels per samples and locus can thereby represent mixtures of distinct epigenomic signatures from different cell types.

To facilitate robust deconvolution of these tumor microenvironment (TME) effects, *Methylayer* uses an unsupervised approach relying on analysis of the cross-correlations between gene expression profiles with promoter methylation signatures.

In broad strokes, *Methylayer*'s normalization strategy is to:

- Compute cross-correlation between gene expression and promoter methylation.
- Cluster the cross-correlation matrix to identify TME expression signatures (i.e. groups of TME genes that affect promoter methylation).
- Use the Euclidean distance in the 2D space of these signatures to identify the K-nearest neighbors of each tumor.
- Subtract from the raw methylation value of each tumor the mean methylation of its K neighbors.

It should be noted that using smaller K values will increase noise (since the neighborhood mean methylation will becomes less stable), while using larger K values may lead to less effective normalization of the TME signatures.

See `?deconv_TME` for more details.

Normalize ER+/ER-/normal TCGA-BRCA samples

```
ER_positive_norm_meth <- deconv_TME(tcga_prom_meth_positive, tcga_expr_pos
itive, all_meth_positive, k = 15) %cache_rds% here("data/TCGA-BRCA/TCGA_BR
CA_ER_positive_norm_meth.rds")

ER_negative_norm_meth <- deconv_TME(tcga_prom_meth_negative, tcga_expr_neg
ative, all_meth_negative, k = 15) %cache_rds% here("data/TCGA-BRCA/TCGA_BR
CA_ER_negative_norm_meth.rds")

normal_norm_meth <- deconv_TME(tcga_prom_meth_normal, tcga_expr_normal, al
l_meth_normal, k = 15) %cache_rds% here("data/TCGA-BRCA/TCGA_BRCA_normals_
norm_meth.rds")
```

Merge all normalized methylation

```
all_norm_meth <- cbind(ER_positive_norm_meth$norm_meth, ER_negative_norm_m
eth$norm_meth, normal_norm_meth$norm_meth) %>% mat_to_intervs()

dim(all_norm_meth)

## [1] 394182    540

# gtrack.rm("TCGA.BRCA_450k_norm", force=TRUE)
if (!gtrack.exists("TCGA.BRCA_450k_norm")) {
  data.table::fwrite(all_norm_meth, here("data/TCGA-BRCA/TCGA_BRCA_all_nor
m_meth.tsv"), na = "nan", row.names = FALSE, quote = FALSE, sep = "\t", sc
ipen = 50)
  gtrack.array.import("TCGA.BRCA_450k_norm", "TME normalized TCGA-BRCA met
hylation", here("data/TCGA-BRCA/TCGA_BRCA_all_norm_meth.tsv"))
}
```

Merge all TME expression scores

```
tme_df <- bind_rows(
  ER_positive_norm_meth$tme_features,
  ER_negative_norm_meth$tme_features,
  normal_norm_meth$tme_features
) %>%
  select(samp, caf, immune, caf.meth, immune.meth) %cache_df%
  here("data/TCGA-BRCA/TCGA_BRCA_TME_features.tsv")

head(tme_df)

##             samp         caf       immune  caf.meth immune.meth
## 1 TCGA_A1_A0SB_T 1.222652536 -1.932360349 0.4297221   0.6180962
## 2 TCGA_A1_A0SE_T 0.585128289  0.009182071 0.5917645   0.7026801
## 3 TCGA_A1_A0SF_T 0.277531944  0.793479727 0.5607568   0.6241409
## 4 TCGA_A1_A0SG_T 0.003446965  0.314856974 0.5603409   0.6285214
## 5 TCGA_A1_A0SI_T 0.621719992  0.757864580 0.5391400   0.6103056
## 6 TCGA_A1_A0SJ_T 0.391526312  0.075408753 0.6324559   0.7086122
```

Diagnose TME normalization

We will extract the correlation of the raw and normalized methylation to gene expression of selected genes in order to see that our normalization worked. Gene expression associations of CAV1, a canonical CAF gene, and CD3D, a canonical T-cell gene have been normalized while cancer-relevant genes such as GATA3 and TOP2A were not affected by our normalization

```
before_after_df <- calc_gene_cor_before_after_deconv(ER_positive_norm_meth
, all_meth_positive %>% mat_to_intervs(), c("CAV1", "CD3D", "GATA3", "TOP2
A")) %>% as_tibble()

before_after_df <- before_after_df %>%
  select(-ends_with(".norm")) %>%
  gather("gene", "cor_raw", -(chrom:end)) %>%
  left_join(before_after_df %>% select(chrom:end, ends_with(".norm")) %>%
gather("gene", "cor_norm", -(chrom:end)) %>% mutate(gene = gsub(".norm$",
"", gene)))

## Joining, by = c("chrom", "start", "end", "gene")

options(repr.plot.width = 8, repr.plot.height = 8)

lims <- c(-0.7, 0.7)

p_before_after <- before_after_df %>%
  mutate(ER = "ER+") %>%
  mutate(
    cor_raw = tgutil::clip_vals(cor_raw, lims[1], lims[2]),
    cor_norm = tgutil::clip_vals(cor_norm, lims[1], lims[2])
  ) %>%
  ggplot(aes(x = cor_raw, y = cor_norm, color = ER)) +
  geom_point(size = 0.001) +
  scale_color_manual(values = annot_colors$ER1) +
  geom_abline(linetype = "dashed") +
  xlab("Raw methylation vs. expression correlation") +
  ylab("Normalized methylation\nvs. expression correlation") +
  facet_wrap(. ~ gene, nrow = 2) +
  guides(color = FALSE) +
  xlim(lims[1], lims[2]) +
  ylim(lims[1], lims[2]) +
  theme(aspect.ratio = 1)

p_before_after + theme_bw() + theme(aspect.ratio = 1)
```

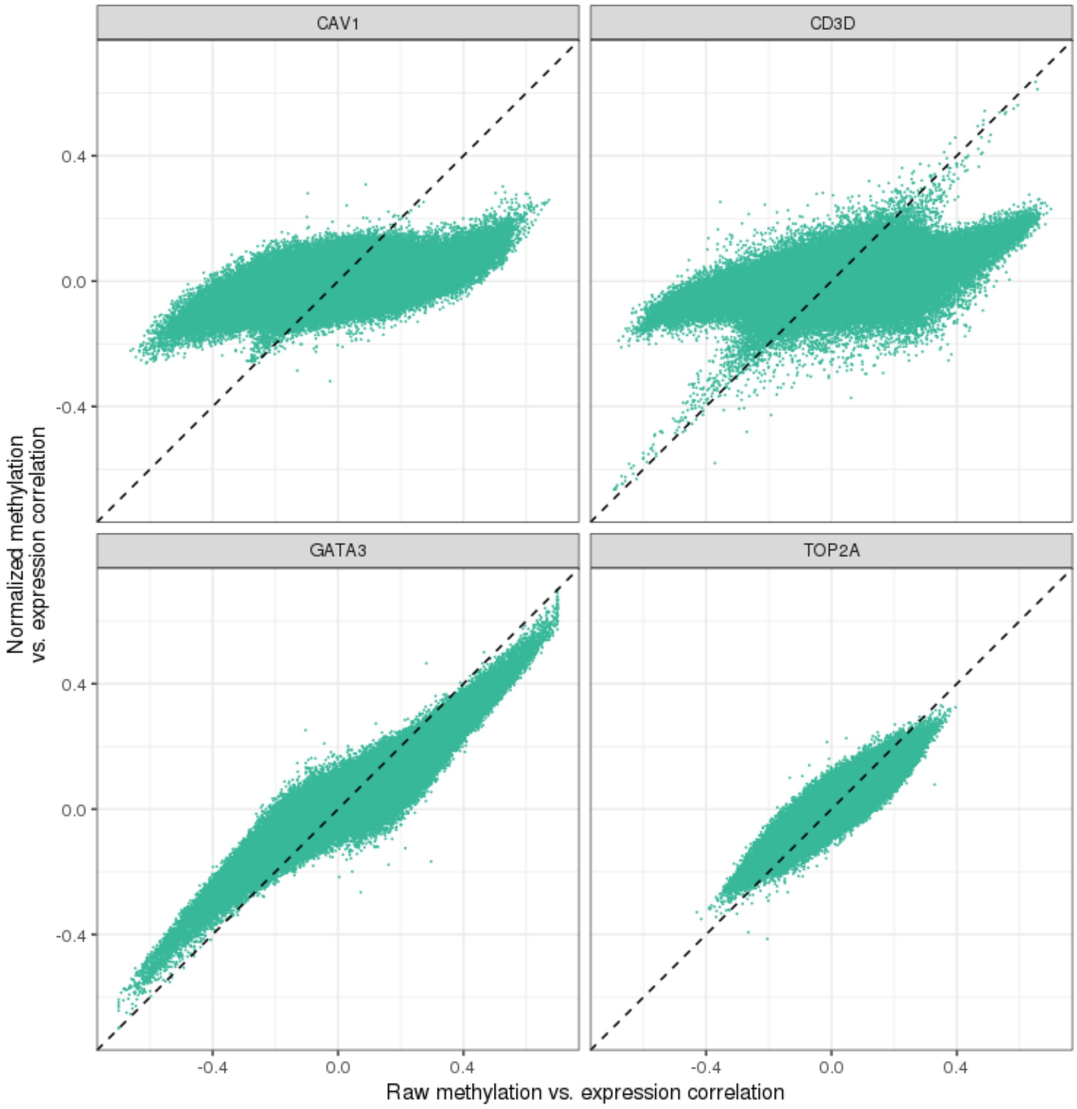

See appendix 1 for more TME normalization diagnostics.

Define epigenomic scores

Now that we have TME-normalized methylation profiles we can look at their correlation structure in order to identify the epigenomic scores. We will start by clustering the normalized methylation of ER+ samples:

```
ER_positive_mat_raw <- all_meth[, intersect(colnames(all_meth), tcga_ER_po
sitive_samples)]

ER_positive_mat <- all_norm_meth %>%
  select(chrom:end, any_of(tcga_ER_positive_samples)) %>%
  intervs_to_mat()
```

Filter loci that have low methylation (average of under 0.1):

```
means <- rowMeans(all_meth, na.rm = TRUE)
means_ER_positive <- rowMeans(ER_positive_mat_raw, na.rm = TRUE)
```

```
meth_thresh <- 0.1

options(repr.plot.width = 4, repr.plot.height = 4)
tibble(m = means_ER_positive) %>% ggplot(aes(x = m)) +
  geom_density() +
  geom_vline(xintercept = meth_thresh) +
  theme_bw()
```

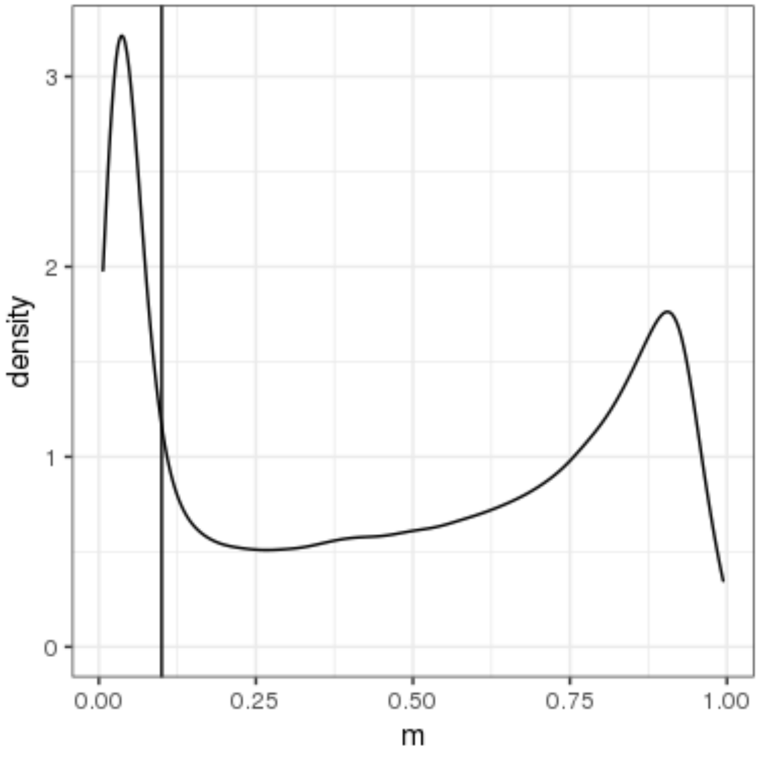


```
ER_positive_mat_s <- ER_positive_mat[means_ER_positive >= meth_thresh, ]
nrow(ER_positive_mat_s)

## [1] 289121
```

We sample 20k loci and calculate a correlation matrix of their methylation values in ER+ samples:

```
set.seed(17)
ER_positive_mat_s <- ER_positive_mat_s[sample(1:nrow(ER_positive_mat_s), 2
e4), ]

cm <- tgs_cor(t(ER_positive_mat_s), pairwise.complete.obs = TRUE) %fcache_
rds% here("data/TCGA-BRCA/TCGA_BRCA_ER_positive_loci_cm_samp.rds")
```

We remove rows and columns without at least one correlation value above 0.25:

```
cm1 <- cm
diag(cm1) <- NA
cor_maxs <- matrixStats::rowMaxs(abs(cm1), na.rm = TRUE)
f <- cor_maxs >= 0.25
f <- f & rowSums(is.na(cm)) == 0
cm_f <- cm[f, f]
```

```
dim(cm_f)

## [1] 19994 19994
```

We cluster the correlation matrix using *hclust*:

```
hc_meth <- as.dist(1 - cm_f) %>% fastcluster::hclust(method = "ward.D2") %
fcache_rds% here("data/TCGA-BRCA/TCGA_BRCA_ER_positive_loci_cm_hclust.rds"
)
```

Reorder the dendrogram according to average methylation:

```
hc_meth <- reorder(hc_meth, rowMeans(ER_positive_mat_raw[rownames(cm_f), ]
, na.rm = TRUE))

k <- 14

options(repr.plot.width = 8, repr.plot.height = 8)
plot_meth_mat_cm(
  cm_f,
  k = k,
  width = 1000,
  height = 1000,
  hc_meth = hc_meth,
  downscale = TRUE,
  zlim = c(-0.3, 0.3),
  colors = c("black", "darkred", "white", "darkblue", "cyan")
)

## downscaling matrix

## downscale k: 10

## plotting
```

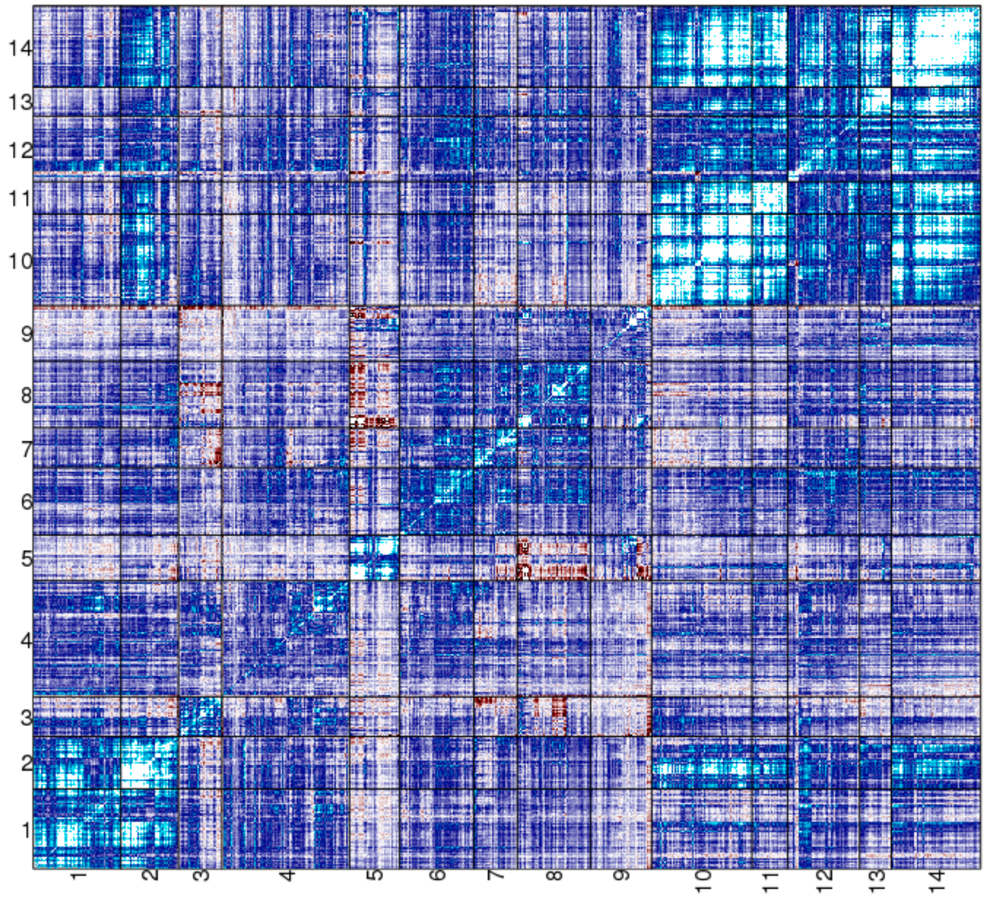

We can see that there is a large group of correlated loci at the top right (10-14), another group in the middle (6-8), and another on at the bottom left (1,2).

Below we compare these clusters to the methylation layers we derived from the METABRIC cohort, and therefore we would call the top right cluster ‘clock’, the middle cluster ‘ML’ and the bottom ‘MG’.

In addition, we have another 2 small clusters (3 and 5) which we term ‘other1’ and ‘other2’ respectively. The other clusters (4,9) look weak in their intra correlation. We combine these and call them ‘no cor’:

```
ct <- cutree_order(hc_meth, k = k)
ct_new <- case_when(
  ct %in% 10:14 ~ "clock",
  ct %in% 6:9 ~ "ML",
  ct %in% 1:2 ~ "MG",
  ct == 3 ~ "other1",
  ct == 5 ~ "other2",
  ct %in% c(4, 9) ~ "no_cor"
)
names(ct_new) <- names(ct)
```

We will generate a score for each tumor based on the mean methylation of each group:

```
feats_mat <- tgs_matrix_tapply(all_norm_meth %>% intervs_to_mat() %>% .[na
mes(ct_new), ] %>% t(), ct_new, mean, na.rm = TRUE) %>% t()

# We add the TME features for comparison
tme_df <- fread(here("data/TCGA-BRCA/TCGA_BRCA_TME_features.tsv")) %>% as_
tibble()
feats_mat <- cbind(feats_mat, tme_df %>% select(samp, caf, immune, caf.met
h, immune.meth) %>% as.data.frame() %>% column_to_rownames("samp"))

feats_df <- feats_mat %>%
  as.data.frame() %>%
  rownames_to_column("samp") %>%
  select(-other1, -other2, -no_cor) %>%
  left_join(tcga_samp_data %>% select(samp = samp_id, ER), by = "samp") %>
%
  select(samp, ER, everything()) %fcache_df%
  here("data/TCGA-BRCA/TCGA_BRCA_epigenomic_features.tsv") %>%
  as_tibble()

head(feats_df)

## # A tibble: 6 x 9
##   samp      ER        clock        MG       ML      caf    immune caf.meth im
mune.meth
##   <chr>     <chr>     <dbl>     <dbl>    <dbl>    <dbl>     <dbl>    <dbl>
<dbl>
## 1 TCGA_A1… ER+    0.0189   -1.25e-1 -0.0958 1.22    -1.93       0.430
0.618
## 2 TCGA_A1… ER+   -0.0194   -2.29e-2  0.0183 0.585    0.00918    0.592
0.703
## 3 TCGA_A1… ER+   -0.00529   3.98e-4 -0.0119 0.278    0.793      0.561
0.624
## 4 TCGA_A1… ER+   -0.0801   -1.00e-1 -0.0266 0.00345  0.315      0.560
0.629
## 5 TCGA_A1… ER+   -0.0542   -3.70e-2 -0.0208 0.622    0.758      0.539
0.610
## 6 TCGA_A1… ER+    0.0664    5.48e-2  0.0235 0.392    0.0754     0.632
0.709
```

Compare TCGA-BRCA epigenomic modules to METABRIC

Since TCGA-BRCA data is 450k based while METABRIC data is RRBS, it would be hard to compare the epigenomic modules directly. Fortunately, we can use the expression data as an anchor and compare the correlations of the methylation modules in each dataset with gene expression. If the genes that are correlated with each module are the same in both datasets it gives us high confidence that we are observing the same effect.

Calculate correlation between the modules and every gene:

```
tcga_gene_cors <- plyr::ddply(feats_df, "ER", function(x) {
  samples <- reduce(list(tcga_samp_data$samp_id, colnames(tcga_expr), x$sa
mp), intersect)
  feats_mat <- x %>%
    select(-ER) %>%
    as.data.frame() %>%
    column_to_rownames("samp") %>%
    as.matrix()
  cm <- tgs_cor(t(tcga_expr[, samples]), feats_mat[samples, ], pairwise.co
mplete.obs = TRUE)
  cm <- cm %>%
    as.data.frame() %>%
    rownames_to_column("name") %>%
    as_tibble()
  return(cm %>% mutate(ER = x$ER[1]))
})
head(tcga_gene_cors)
```

```
##    name       clock           MG          ML         caf       immune
## 1  A1BG  0.04849510  0.003149984  0.24601114  0.04908175 -0.074749797
## 2  A1CF  0.10798897  0.061904818 -0.03467173 -0.01548953  0.110957541
## 3 A2BP1  0.01341289 -0.000587340  0.22526708  0.19762620  0.007679025
## 4 A2LD1 -0.04122864 -0.097998921  0.01634378  0.09301897  0.088271914
## 5 A2ML1 -0.13092742 -0.189658881 -0.33538301 -0.03703322  0.039981388
## 6   A2M  0.01943741 -0.128176602 -0.10856015  0.44829072  0.196500885
##      caf.meth  immune.meth  ER
## 1  0.08028370  0.114162819 ER-
## 2 -0.01249476 -0.045891226 ER-
## 3 -0.07261832 -0.009398886 ER-
## 4 -0.06744866 -0.052208571 ER-
## 5 -0.10419845 -0.186983947 ER-
## 6 -0.19331311 -0.152809835 ER-
```

```
metabric_gene_cors <- get_expression_features_cors() %>% mutate(clock = -c
lock, ML = -ML)
```

Note that the METABRIC scores that involved loss of methylation in tumors were reversed (so that higher score => higher difference from the normal), so we reverse them back in order to compare with the TCGA-BRCA modules

```
df <- tcga_gene_cors %>%
  rename(
    tcga.clock = clock,
    tcga.MG = MG,
    tcga.ML = ML,
```

```
    tcga.caf = caf,
    tcga.immune = immune,
    tcga.caf.meth = caf.meth,
    tcga.immune.meth = immune.meth
  ) %>%
  left_join(metabric_gene_cors, by = c("name", "ER")) %>%
  mutate(ER = factor(ER, levels = c("ER+", "ER-", "normal")))

df_cross <- df %>%
  gather("feat1", "cor1", -ER, -name) %>%
  left_join(df %>% gather("feat2", "cor2", -ER, -name)) %>%
  filter(grepl("tcga", feat1), !grepl("tcga", feat2)) %>%
  na.omit() %>%
  filter(is.finite(cor1), is.finite(cor2)) %>%
  group_by(feat1, feat2, ER) %>%
  mutate(feat_cor = cor(cor1, cor2, use = "pairwise.complete.obs"))

## Joining, by = c("name", "ER")

options(repr.plot.width = 10, repr.plot.height = 10)
df_cross1 <- df_cross %>%
  filter(ER == "ER+") %>%
  filter(!grepl("immune", feat1), !grepl("caf", feat1), !grepl("immune", f
eat2), !grepl("caf", feat2))
df_cross1 %>%
  ggplot(aes(x = cor1, y = cor2, color = ER)) +
  geom_point(size = 0.5) +
  theme_bw() +
  theme(aspect.ratio = 1) +
  facet_wrap(~ER) +
  scale_color_manual(values = annot_colors$ER1) +
  geom_label(data = df_cross1 %>% distinct(feat1, feat2, feat_cor, ER) %>%
mutate(feat_cor = paste0("cor = ", round(feat_cor, digits = 2))), inherit.
aes = TRUE, aes(label = feat_cor), x = -0.2, y = 0.2, color = "black") +
  facet_grid(feat2 ~ feat1, scales = "free")
```

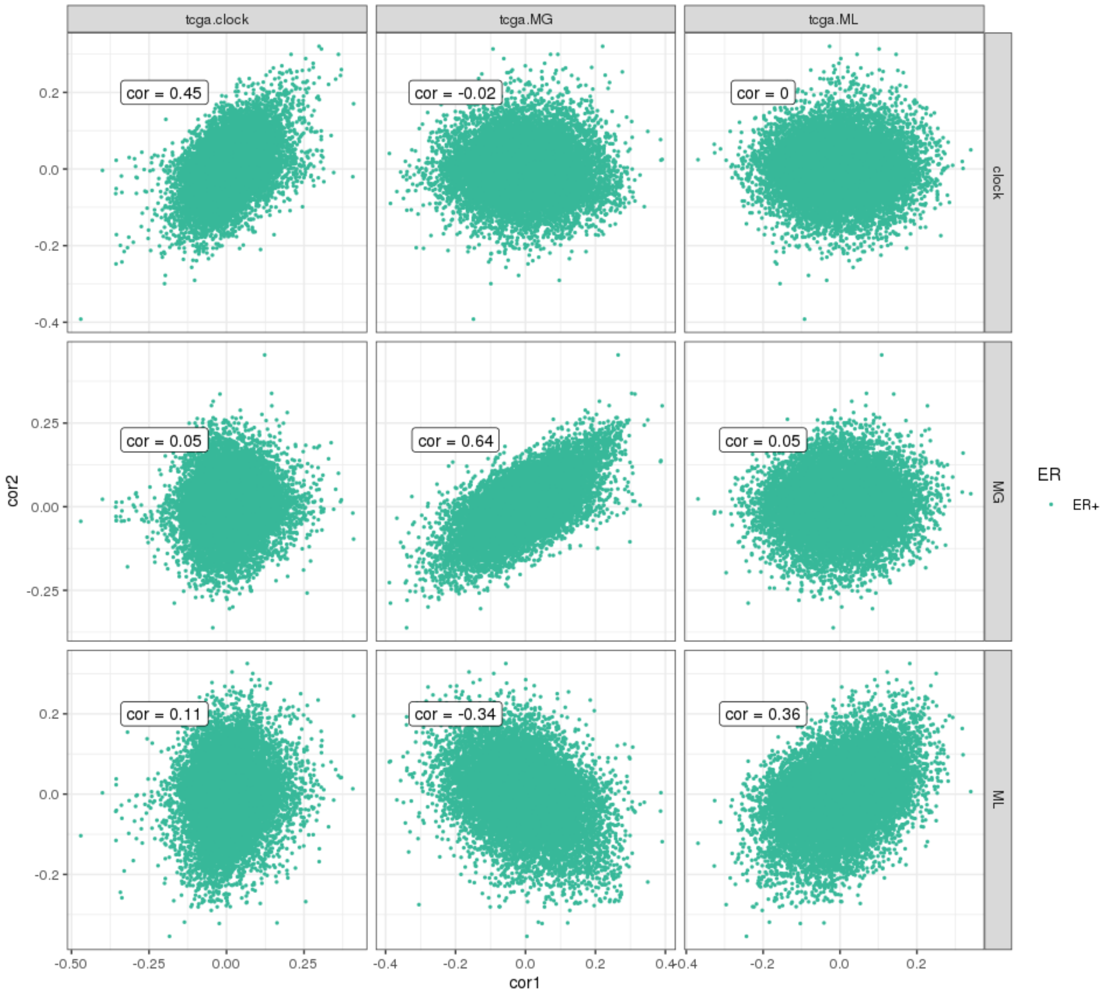

X axis has the correlations of gene expression of 17,125 genes with the 3 modules we identified in the TCGA-BRCA dataset and Y axis has correlations of the same genes with the 3 METABRIC modules.

We can see indeed that there is a good correspondence between the modules we termed "clock", "MG" and "ML" in the TCGA dataset and the same modules in the METABRIC cohort.

We will now plot the same correlations for ER- tumors:

```
options(repr.plot.width = 10, repr.plot.height = 10)
df_cross1 <- df_cross %>%
  filter(ER == "ER-") %>%
  filter(!grepl("immune", feat1), !grepl("caf", feat1), !grepl("immune", f
eat2), !grepl("caf", feat2))
df_cross1 %>%
  ggplot(aes(x = cor1, y = cor2, color = ER)) +
  geom_point(size = 0.5) +
  theme_bw() +
  theme(aspect.ratio = 1) +
  facet_wrap(~ER) +
```

```
  scale_color_manual(values = annot_colors$ER1) +
  geom_label(data = df_cross1 %>% distinct(feat1, feat2, feat_cor, ER) %>%
mutate(feat_cor = paste0("cor = ", round(feat_cor, digits = 2))), inherit.
aes = TRUE, aes(label = feat_cor), x = -0.2, y = 0.2, color = "black") +
  facet_grid(feat2 ~ feat1, scales = "free")
```

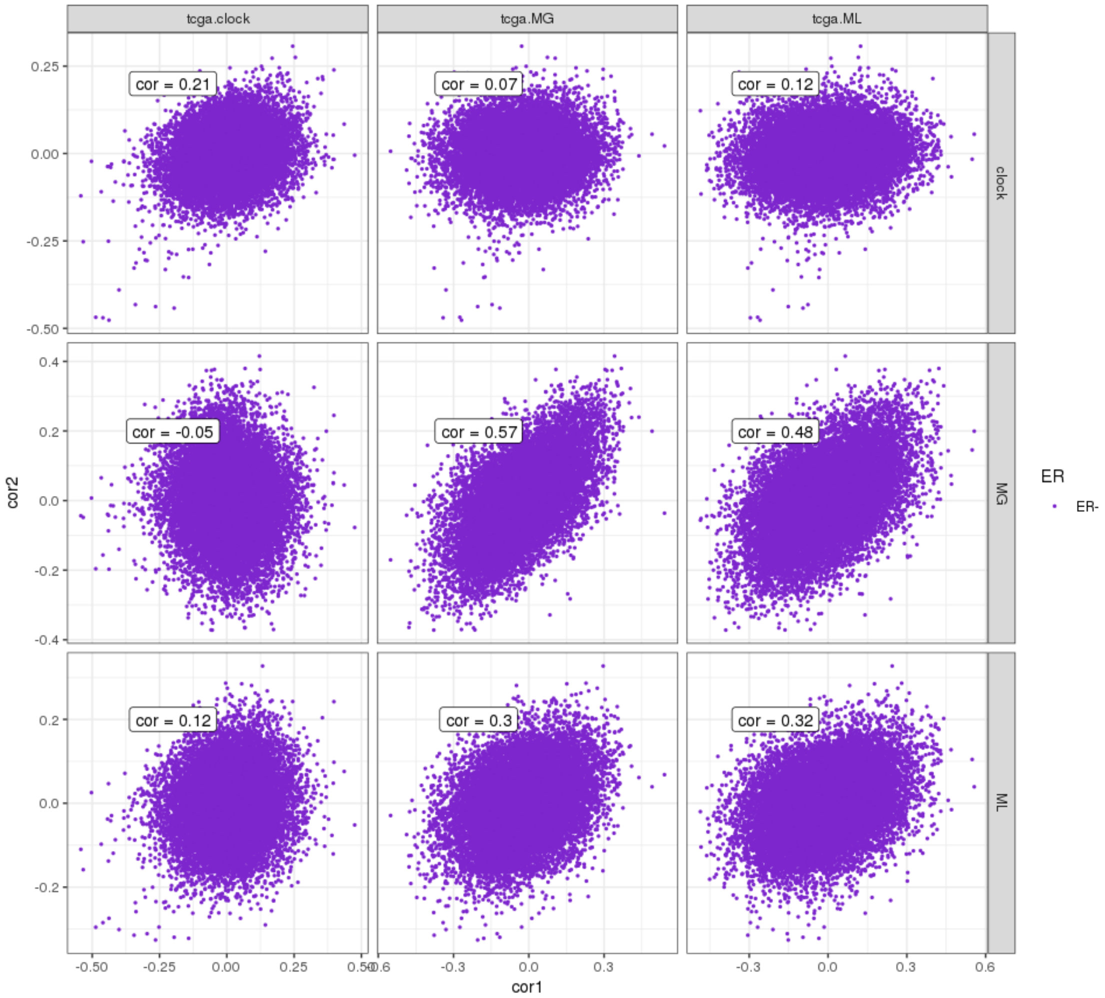

We can see that the correlation exists also in the ER- tumors, though to a less extent. Interestingly, MG and ML in ER- TCGA tumors are both correlated to MG and ML in METABRIC (MG to ML and vice versa), and indeed a similar correlation is seen in ER- METABRIC samples (Figure 2E, bottom).

Correlation between the features

We look at the correlations between the features in TCGA ER+/ER-/normal samples separately:

```
options(repr.plot.width = 10, repr.plot.height = 10)
GGally::ggpairs(feats_mat[intersect(rownames(feats_mat), tcga_ER_positive_
samples), ] %>% as.data.frame(), progress = FALSE) + theme_bw()
```

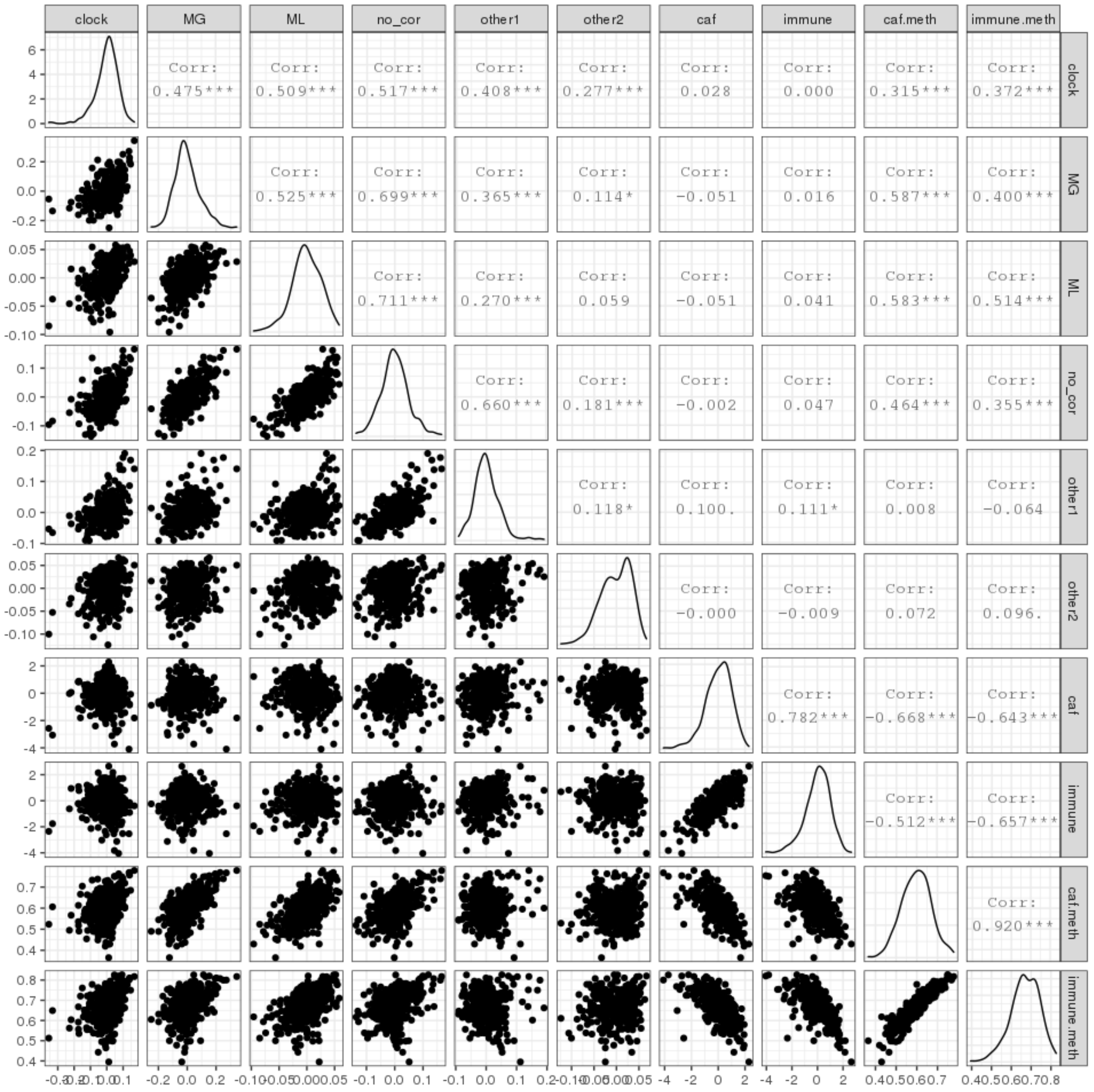


```r
options(repr.plot.width = 7, repr.plot.height = 7)
feats_cm <- tgs_cor(as.matrix(feats_mat), pairwise.complete.obs = TRUE)
gather_matrix(feats_cm) %>%
  mutate(val = round(val, digits = 2)) %>%
  mutate(x = factor(x, levels = c("clock", "ML", "MG", "other1", "other2",
"no_cor", "immune", "caf"))) %>%
  mutate(y = factor(y, levels = c("clock", "ML", "MG", "other1", "other2",
"no_cor", "immune", "caf"))) %>%
  filter(!is.na(x), !is.na(y)) %>%
  ggplot(aes(x = x, y = y, fill = val, label = val)) +
  geom_tile() +
  geom_text() +
  scale_fill_gradient2(low = "blue", high = "red") +
  theme_bw() +
  theme(aspect.ratio = 1)
```

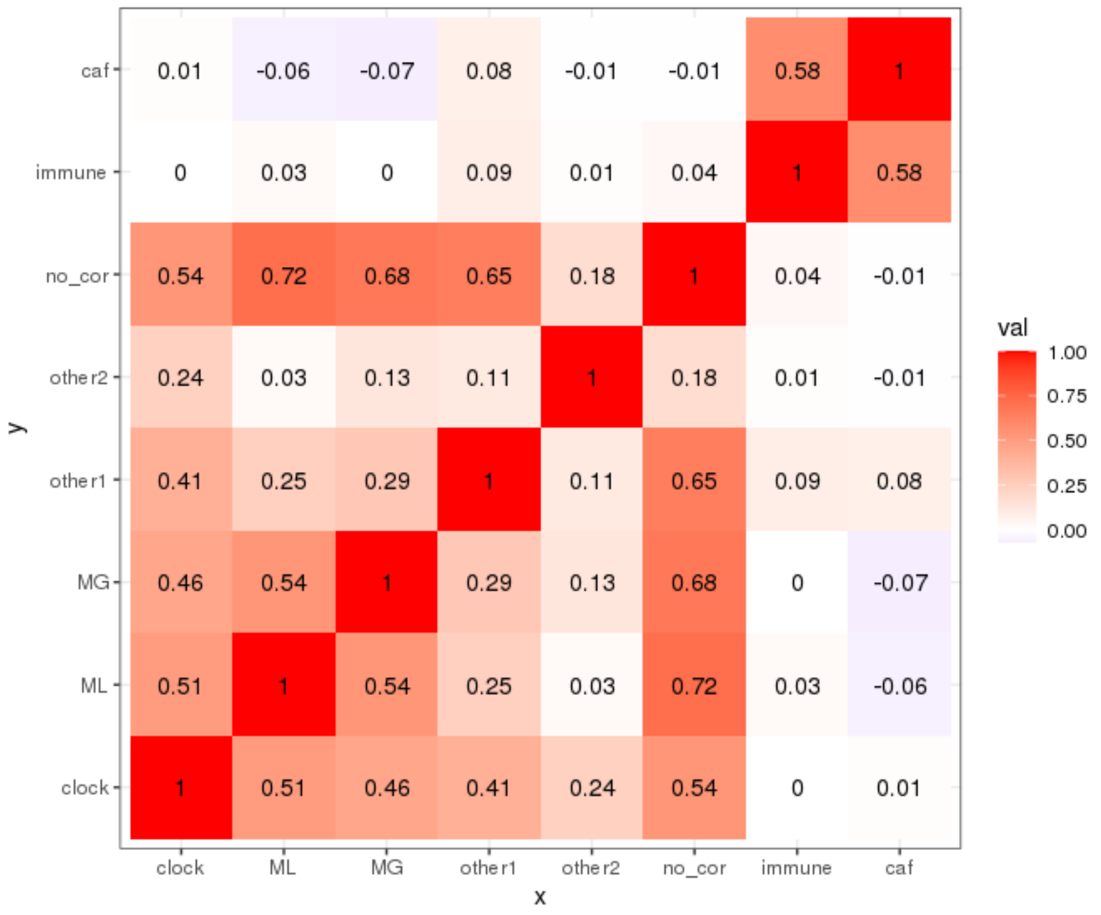


```
options(repr.plot.width = 10, repr.plot.height = 10)
GGally::ggpairs(feats_mat[intersect(rownames(feats_mat), tcga_ER_negative_
samples), ] %>% as.data.frame(), progress = FALSE) + theme_bw()
```

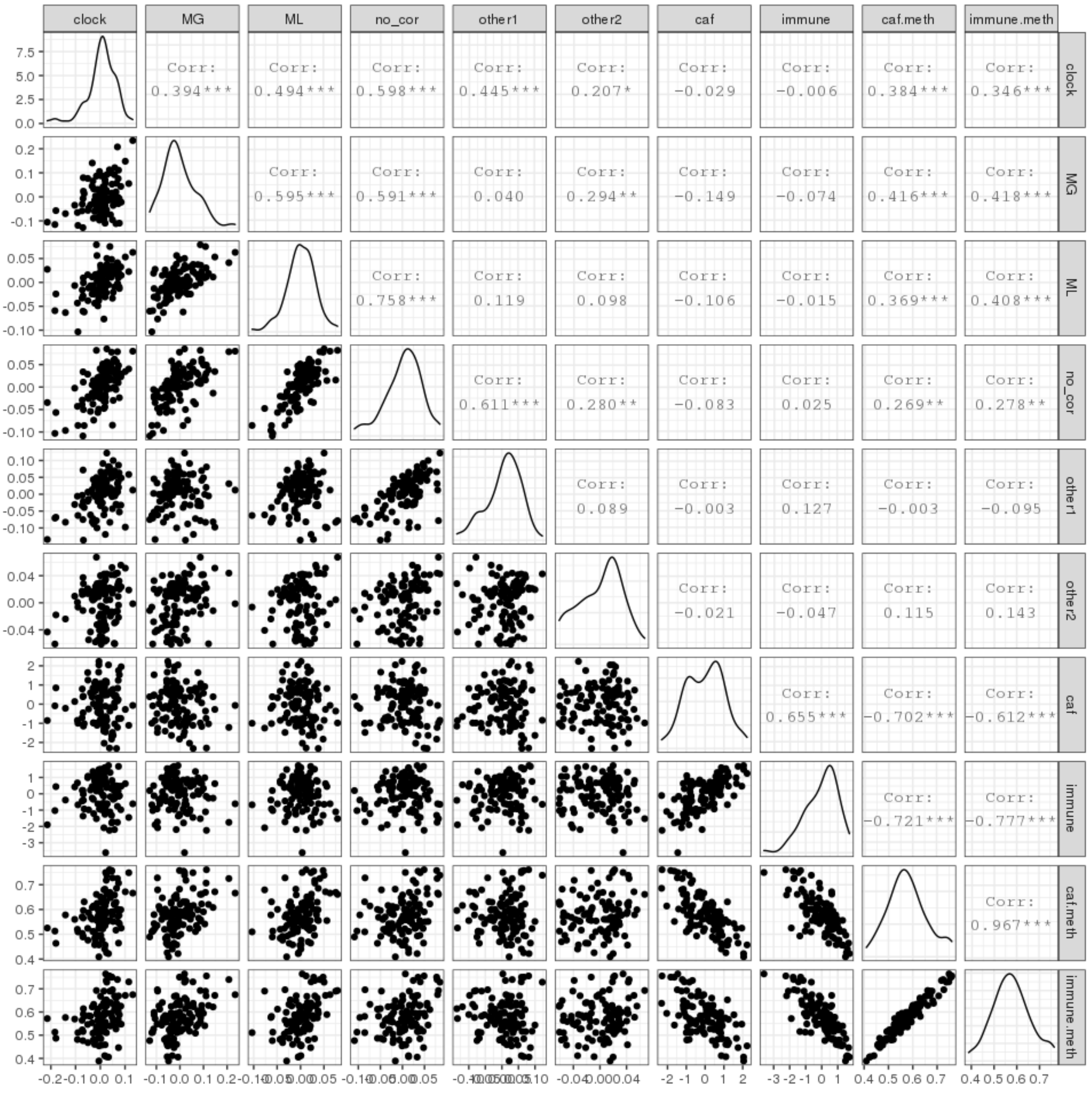


```
options(repr.plot.width = 10, repr.plot.height = 10)
GGally::ggpairs(feats_mat[intersect(rownames(feats_mat), tcga_normal_sampl
es), ] %>% as.data.frame(), progress = FALSE) + theme_bw()
```

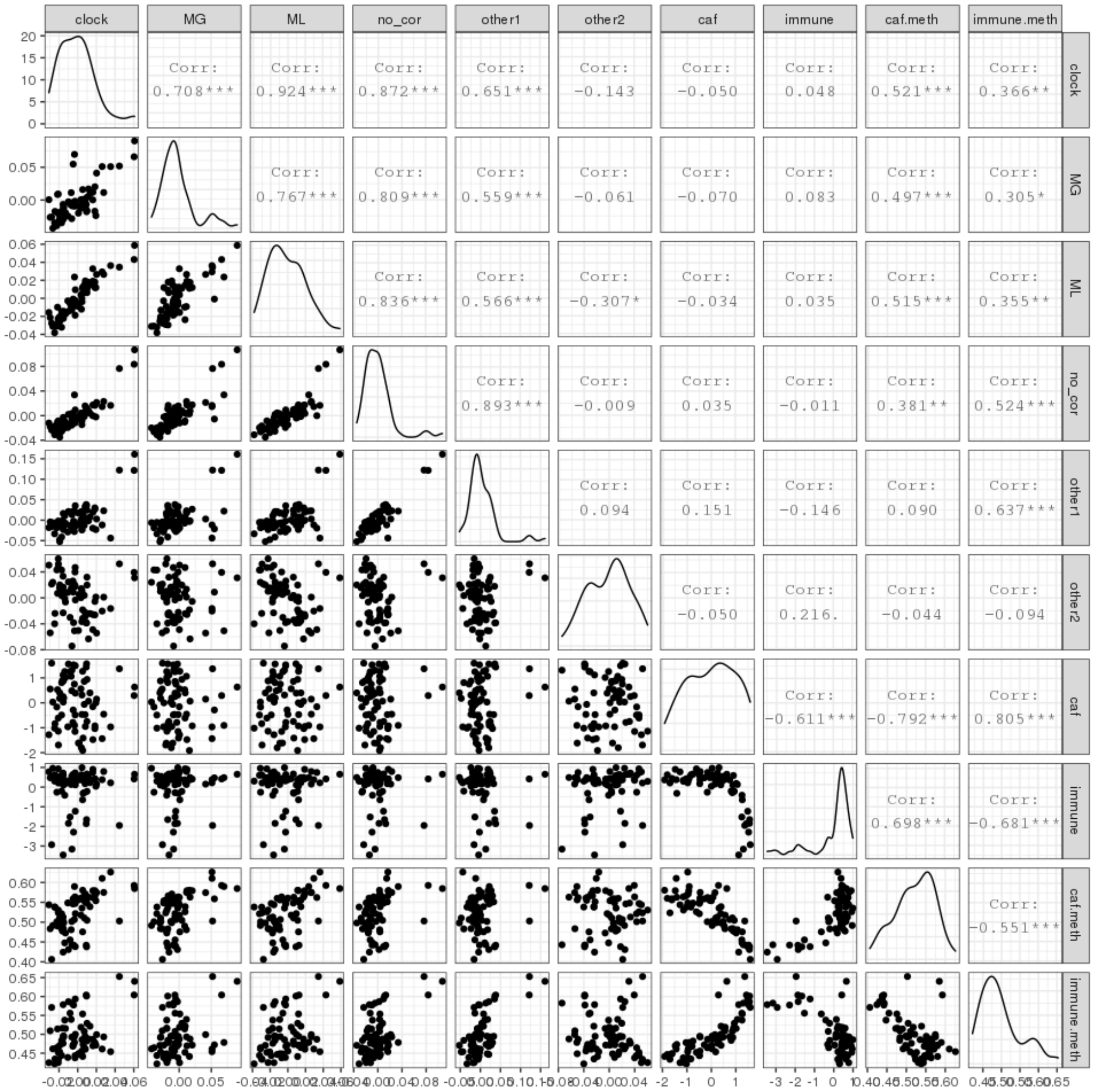

Methylation regulation of gene expression *in cis*

We start by identifying genes that are strongly correlated to TME (immune and CAF) in the expression-methylation correlation clusters. Our TME normalization cleaned most of their correlations with methylation, thereby enhancing the more interesting expression-methylation in-cis correlations.

```
TME_genes <-
  {
    ER_pos_TME_genes <- get_TME_genes(readr::read_rds(here("data/TCGA-BRCA
/TCGA_BRCA_ER_positive_norm_meth.rds"))$em_cross_clust)
    ER_neg_TME_genes <- get_TME_genes(readr::read_rds(here("data/TCGA-BRCA
/TCGA_BRCA_ER_negative_norm_meth.rds"))$em_cross_clust)
    normal_TME_genes <- get_TME_genes(readr::read_rds(here("data/TCGA-BRCA
/TCGA_BRCA_normals_norm_meth.rds"))$em_cross_clust)

    unique(c(ER_pos_TME_genes, ER_neg_TME_genes, normal_TME_genes))
  } %cache_rds% here("data/TCGA-BRCA/TCGA_BRCA_TME_genes.rds")
```

```
length(TME_genes)

## [1] 2421

expr_mat_f <- tcga_expr[!(rownames(
  tcga_expr
) %in% TME_genes), ]
dim(expr_mat_f)

## [1] 18080  1212
```

Load normalized methylation and separate it to promoters and non-promoters

```
prom_meth <- get_tcga_brca_prom_meth_TME_norm() %>% mat_to_intervs()

non_prom_meth <- get_tcga_brca_genomic_meth(track = "TCGA.BRCA_450k_norm")
%>% mat_to_intervs()

prom_intervs_f <- resolve_alt_promoters(prom_meth %>% select(chrom:end))
```

Promoters

We use *Methylayer* to identify promoters that are correlated in cis to the expression of their gene. We start by creating matrices with promoter methylation for ER+/ER-/normal samples.

```
ER_positive_prom_mat_norm <- prom_meth %>%
  select(chrom:end, any_of(tcga_ER_positive_samples)) %>%
  intervs_to_mat()
ER_negative_prom_mat_norm <- prom_meth %>%
  select(chrom:end, any_of(tcga_ER_negative_samples)) %>%
  intervs_to_mat()
normal_prom_mat_norm <- prom_meth %>%
  select(chrom:end, any_of(tcga_normal_samples)) %>%
  intervs_to_mat()

dim(ER_positive_prom_mat_norm)

## [1] 32378   357

dim(ER_negative_prom_mat_norm)

## [1] 32378   111

dim(normal_prom_mat_norm)

## [1] 32378    69
```

We remove rows with missing values (these are loci that were not covered by 450k arrays).

```
f <- rowSums(is.na(ER_positive_prom_mat_norm)) == 0 & rowSums(is.na(ER_neg
ative_prom_mat_norm)) == 0 & rowSums(is.na(normal_prom_mat_norm)) == 0
sum(f)

## [1] 24670
```

We then use the function *cis_em_promoters* to detect promoters that are correlated to their gene expression *in cis*. The approach *Methylayer* is taking is relatively simple:

1. Compute the expression-methylation correlation for each promoter and each gene.
2. For every gene, rank its correlations with promoters, and look at the rank of the gene's own promoter.
3. We then estimate the significance of the rank of the gene's own promoter (see methods). Specifically, if the highest correlation of a gene is with its own promoter there is a high probability that this correlation is specific *in cis* and not a part of a large methylation effects that correlate many loci with multiple genes (*in trans* effect).

```
tcga_prom_cands <- bind_rows(
  cis_em_promoters(ER_positive_prom_mat_norm[f, ], expr_mat_f, prom_interv
s_f, min_samples = 50) %>% mutate(ER = "ER+"),
  cis_em_promoters(ER_negative_prom_mat_norm[f, ], expr_mat_f, prom_interv
s_f, min_samples = 50) %>% mutate(ER = "ER-"),
  cis_em_promoters(normal_prom_mat_norm[f, ], expr_mat_f, prom_intervs_f,
min_samples = 50) %>% mutate(ER = "normal")
) %cache_df% here("data/TCGA-BRCA/TCGA_BRCA_promoter_cis_cands.tsv") %>% a
s_tibble()

df <- tcga_prom_cands %>%
  filter(r == 1) %>%
  distinct(fdr, n_fdr, ER)

df_fdr <- tcga_prom_cands %>%
  filter(fdr < 0.05) %>%
  group_by(ER) %>%
  filter(fdr == max(fdr)) %>%
  distinct(fdr, n_fdr, ER)

df

## # A tibble: 3 x 3
##       fdr n_fdr ER
##     <dbl> <int> <chr>
## 1 0.00115   870 ER+
## 2 0.00186   537 ER-
## 3 0.0385     26 normal

df_fdr

## # A tibble: 3 x 3
## # Groups:   ER [3]
##      fdr n_fdr ER
##    <dbl> <int> <chr>
## 1 0.0498  3193 ER+
```

```
## 2 0.0496  2176 ER-
## 3 0.0385    26 normal
```

```
glue("we identified {n_top_ER_pos} promoters in ER+ and {n_top_ER_neg} in
ER- (FDR<0.01; {n_fdr_ER_pos} in ER+ and {n_fdr_ER_neg} in ER- if increasi
ng FDR to <0.05)",
  n_top_ER_pos = df$n_fdr[df$ER == "ER+"],
  n_top_ER_neg = df$n_fdr[df$ER == "ER-"],
  n_fdr_ER_pos = df_fdr$n_fdr[df_fdr$ER == "ER+"],
  n_fdr_ER_neg = df_fdr$n_fdr[df_fdr$ER == "ER-"]
)
```

```
## we identified 870 promoters in ER+ and 537 in ER- (FDR<0.01; 3193 in ER
+ and 2176 in ER- if increasing FDR to <0.05)
```

Compare to METABRIC *cis* regulated genes

```
metabric_prom_cands <- fread(here("data/promoter_cis_cands.tsv")) %>% as_t
ibble()

max_r <- 1
tcga_top_cands <- tcga_prom_cands %>% filter(r <= max_r)
metabric_top_cands <- metabric_prom_cands %>% filter(r <= max_r)

genes <- intersect(tcga_prom_cands$name, metabric_prom_cands$name)

tcga_top_genes <- tcga_top_cands %>%
  filter(name %in% genes) %>%
  pull(name) %>%
  unique()
metabric_top_genes <- metabric_top_cands %>%
  filter(name %in% genes) %>%
  pull(name) %>%
  unique()

scales::percent(sum(metabric_top_genes %in% tcga_top_genes) / length(metab
ric_top_genes))
```

```
## [1] "53%"
```

```
scales::percent(sum(tcga_top_genes %in% metabric_top_genes) / length(tcga_
top_genes))
```

```
## [1] "40%"
```

```
options(repr.plot.width = 4, repr.plot.height = 4)
grid.draw(list(`METABRIC` = metabric_top_genes, `TCGA` = tcga_top_genes) %
>% VennDiagram::venn.diagram(filename = NULL))
```

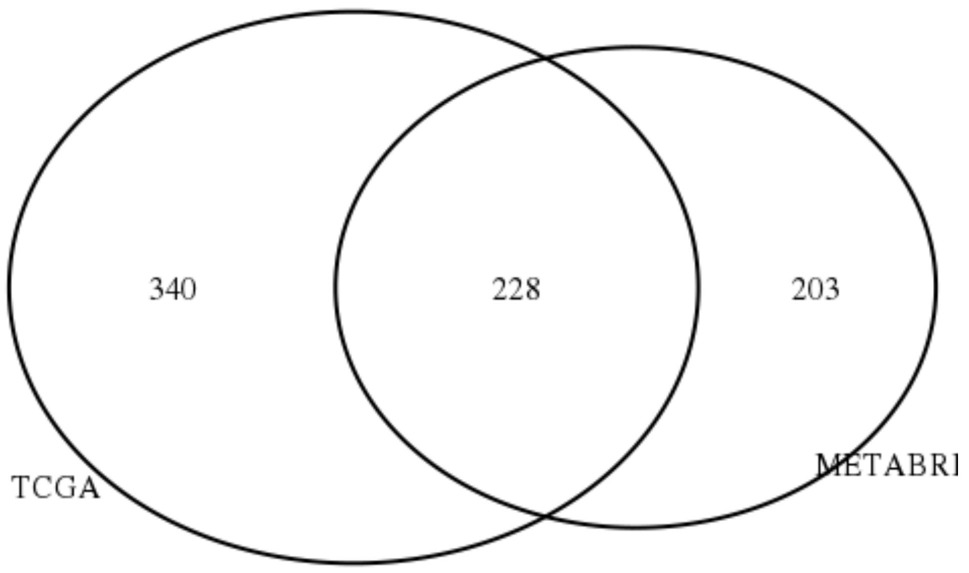

We can see that 53% of the genes that were identified as candidates for cis regulation in the METABRIC cohort were also identified as such in TCGA, and 40% of the TCGA candidate genes were also identified in the METABRIC cohort.

```
phyper(length(intersect(metabric_top_genes, tcga_top_genes)),
length(metabric_top_genes), length(genes) - length(metabric_top_genes),
length(tcga_top_genes), lower.tail=FALSE)
```

```
## [1] 1.228326e-163
```

Non-promoters

We use *Methylayer* to identify non-promoter regions that are correlated *in cis* to expression of *any* gene within their vicinity (within 500kb to the TSS).

Since a locus can be correlated *in cis* with multiple genes, we modify the procedure we used for the promoters and rank the correlation of every locus with **all** the genes, and then examine the ranks of genes that are within 500kb to the locus. We defined a locus as *paired* if the TSS of its top (or k-highest) correlated gene expression profile was within 500kb from it.

We can then estimate the FDR for pairing using the ratio between pairing events in real and shuffled data (we shuffle the correlations of each gene between the loci).

```
ER_positive_genomic_mat <- non_prom_meth %>%
  select(chrom:end, any_of(tcga_ER_positive_samples)) %>%
  intervs_to_mat()
ER_negative_genomic_mat <- non_prom_meth %>%
  select(chrom:end, any_of(tcga_ER_negative_samples)) %>%
  intervs_to_mat()
```

```
normal_genomic_mat <- non_prom_meth %>%
  select(chrom:end, any_of(tcga_normal_samples)) %>%
  intervs_to_mat()
```

```
dim(ER_positive_genomic_mat)
```

```
## [1] 176342    357
```

```
dim(ER_negative_genomic_mat)
```

```
## [1] 176342    111
```

```
dim(normal_genomic_mat)
```

```
## [1] 176342     69
```

```
gene_tss <- get_gene_tss_coord()
```

```
## Joining, by = "full_name"
```

```
genomic_cands_ER_pos <- cis_em_genomic(ER_positive_genomic_mat, expr_mat_f
, gene_tss, min_samples = 50, max_dist = 5e5, min_dist = 200) %>% mutate(E
R = "ER+") %cache_df% here("data/TCGA-BRCA/TCGA_BRCA_genomic_cis_cands_ER_
positive.tsv")
```

```
genomic_cands_ER_neg <- cis_em_genomic(ER_negative_genomic_mat, expr_mat_f
, gene_tss, min_samples = 50, max_dist = 5e5, min_dist = 200) %>% mutate(E
R = "ER-") %cache_df% here("data/TCGA-BRCA/TCGA_BRCA_genomic_cis_cands_ER_
negative.tsv")
```

```
genomic_cands_normals <- cis_em_genomic(normal_genomic_mat, expr_mat_f, ge
ne_tss, min_samples = 50, max_dist = 5e5, min_dist = 200) %>% mutate(ER =
"normal") %cache_df% here("data/TCGA-BRCA/TCGA_BRCA_genomic_cis_cands_norm
al.tsv")
```

```
genomic_cis_cands <- bind_rows(
  genomic_cands_ER_pos,
  genomic_cands_ER_neg,
  genomic_cands_normals
) %>% as_tibble()
head(genomic_cis_cands)
```

```
## # A tibble: 6 x 16
##   chrom  start    end type   rank gene     cor chrom_expr start_expr en
d_expr
##   <chr>  <int>  <int> <chr> <int> <chr>  <dbl> <chr>           <int>
<int>
## 1 chr1   91549  91550 obs       1 SR140 -0.330 <NA>               NA   N
A
## 2 chr1  135251 135252 obs       1 SHRO… -0.221 chr5        132162002
1.32e8
## 3 chr1  530958 530959 obs       1 MCHR2 -0.321 chr6        100442099
1.00e8
## 4 chr1  533949 533950 obs       1 FTHL… -0.390 chrX         31090170
```

```
3.11e7
## 5 chr1  542757 542758 obs       1 ENTP… -0.264 chr14        74486026
7.45e7
## 6 chr1  565169 565170 obs       1 MAT1A -0.214 chr10        82049434
8.20e7
## # … with 6 more variables: strand_expr <int>, dist <int>, n_obs <int>,
## #   n_shuff <int>, fdr <dbl>, ER <chr>
```

```r
dim(genomic_cis_cands)
```

```
## [1] 52878500       16
```

Appendix 1: additional TME normalization diagnostics

Full expression-methylation correlation matrix before normalization

We can look at the cross-correlation matrix (of ER+) before normalization. Rows are genes and columns are methylation profiles. *Methylayer* identified the immune module as module number 5 and the CAFs module as module number 6.

```
options(repr.plot.width = 7, repr.plot.height = 10)
plot_em_cross_cor(ER_positive_norm_meth$em_cross_clust)

## plotting em cross
```

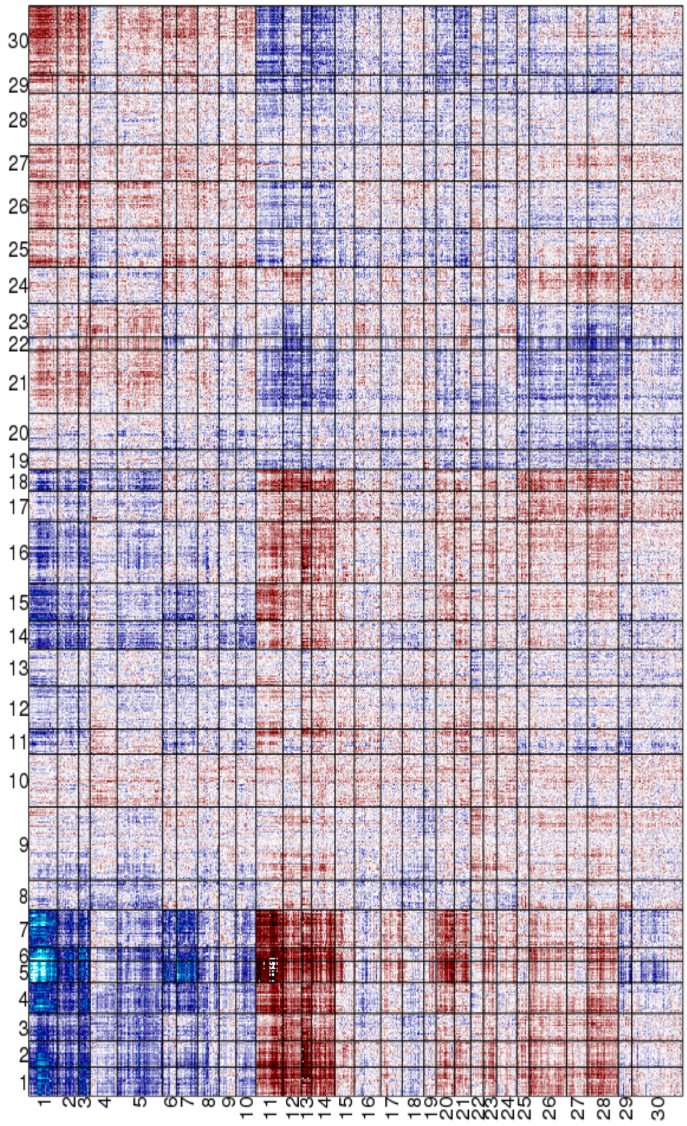

REFERENCES

1. Lutsik, P. *et al.* MeDeCom: discovery and quantification of latent components of heterogeneous methylomes. *Genome Biol.* **18**, 55 (2017).
