# Supplementary material for: DNA methylation landscapes of 1538 breast cancers reveal a replication-linked clock, epigenomic instability and cis-regulation
Source: Nat Commun. 2021 Sep 13;12:5406. doi: 10.1038/s41467-021-25661-w (PMC8437946; doi:10.1038/s41467-021-25661-w)
Supplement: Supplementary file 2 — Description of Additional Supplementary Files [file 41467_2021_25661_MOESM2_ESM.pdf]

Description of Additional Supplementary Files

File Name: Supplementary Data 1
Description: METABRIC samples profiled in this study.

File Name: Supplementary Data 2
Description: Table of genes that have an absolute correlation higher than 0.3 to the Immune expression signature, separated by ER status.

File Name: Supplementary Data 3
Description: Table of genes that have an absolute correlation higher than 0.3 to the CAF expression signature, separated by ER status.

File Name: Supplementary Data 4
Description: Expression-Methylation correlation tables.

File Name: Supplementary Data 5
Description: Table with CAF, Immune, Clock, MG and ML methylation scores per METABRIC sample.

File Name: Supplementary Data 6
Description: Tables of genes that have an absolute expression correlation higher than 0.3 to Clock/ MG/ ML scores.

File Name: Supplementary Data 7
Description: Coordinates of loci that were used in order to calculate the CAF, Immune, Clock, MG and ML scores.

File Name: Supplementary Data 8
Description: Pairs of loci and genes that are candidates for cis regulation at different FDR thresholds. Promoters and genomic loci are shown in separate tables.

File Name: Supplementary Data 9
Description: Table of autosome promoters that show increase of at least 10% in methylation when amplified to at least 3N vs 2N (‘Gain 3N’ table), at least 4N vs 2N (‘Amplification 4N’). The table ‘Loss’ shows loci that showed decreased methylation of at least 10% when losing a copy (1N vs 2N).

File Name: Supplementary Data 10
Description: Table of the list of primers used in the RRBS library preparation.
