# Supplementary material for: DNA methylation landscapes of 1538 breast cancers reveal a replication-linked clock, epigenomic instability and cis-regulation
Source: Nat Commun. 2021 Sep 13;12:5406. doi: 10.1038/s41467-021-25661-w (PMC8437946; doi:10.1038/s41467-021-25661-w)
Supplement: Supplementary file 4 — Reporting Summary [file 41467_2021_25661_MOESM4_ESM.pdf]

nature research

Corresponding author(s): Carlos Caldas
Amos Tanay

Last updated by author(s): Jul 1, 2021

Reporting Summary

Nature Research wishes to improve the reproducibility of the work that we publish. This form provides structure for consistency and transparency in reporting. For further information on Nature Research policies, see our Editorial Policies and the Editorial Policy Checklist.

Statistics

For all statistical analyses, confirm that the following items are present in the figure legend, table legend, main text, or Methods section.

n/a	Confirmed	
☐	☒	The exact sample size (n) for each experimental group/condition, given as a discrete number and unit of measurement
☐	☒	A statement on whether measurements were taken from distinct samples or whether the same sample was measured repeatedly
☐	☒	The statistical test(s) used AND whether they are one- or two-sided<br>*Only common tests should be described solely by name; describe more complex techniques in the Methods section.*
☐	☒	A description of all covariates tested
☐	☒	A description of any assumptions or corrections, such as tests of normality and adjustment for multiple comparisons
☐	☒	A full description of the statistical parameters including central tendency (e.g. means) or other basic estimates (e.g. regression coefficient) AND variation (e.g. standard deviation) or associated estimates of uncertainty (e.g. confidence intervals)
☐	☒	For null hypothesis testing, the test statistic (e.g. F, t, r) with confidence intervals, effect sizes, degrees of freedom and P value noted<br>*Give P values as exact values whenever suitable.*
☒	☐	For Bayesian analysis, information on the choice of priors and Markov chain Monte Carlo settings
☒	☐	For hierarchical and complex designs, identification of the appropriate level for tests and full reporting of outcomes
☐	☒	Estimates of effect sizes (e.g. Cohen's d, Pearson's r), indicating how they were calculated

Our web collection on statistics for biologists contains articles on many of the points above.

Software and code

Policy information about availability of computer code

Data collection	No software used
Data analysis	All analysis code is available for academic use at https://github.com/tanaylab/metabric_rrbs.<br><br>Bismark suite (version 0.13.1) (Krueger, F. & Andrews, S. R. et al., 2011)<br>All analysis was done with R 4.0.3<br>methylayer package (developed as part of manuscript, https://github.com/tanaylab/methylayer)<br>In-house gpatterns package (version 0.2, https://github.com/tanaylab/gpatterns)<br>umap package (version 0.2.2.0, https://cran.r-project.org/web/packages/umap/index.html)<br>survminer package (version 0.4.8, https://cran.r-project.org/web/packages/survminer/index.html)

For manuscripts utilizing custom algorithms or software that are central to the research but not yet described in published literature, software must be made available to editors and reviewers. We strongly encourage code deposition in a community repository (e.g. GitHub). See the Nature Research guidelines for submitting code & software for further information.

Data

Policy information about availability of data

All manuscripts must include a data availability statement. This statement should provide the following information, where applicable:
- Accession codes, unique identifiers, or web links for publicly available datasets
- A list of figures that have associated raw data
- A description of any restrictions on data availability

All primary RRBS data (fastq files) and raw methylation calls (hg19 and hg38) are deposited at the European Genome-phenome Archive (EGA) under study accession

number: EGAS00001004327 [https://ega-archive.org/studies/EGAS00001004327]. Data can be downloaded upon request to EGA (through the METABRIC Data Access Committee). Processed promoter and genomic DNA methylation values (raw and normalized) for the 1538 tumor samples and 244 adjacent normal tissues are available at http://www.wisdom.weizmann.ac.il/~atanay/metabric/methylation_profiles.tar.gz.

The genomic copy number, gene-expression, somatic mutation and molecular-subtype information has been described previously6,7 and is available at the European Genome-phenome Archive (EGAS00000000083 [https://ega-archive.org/studies/EGAS00000000083]).

The TCGA BRCA methylation 450k dataset is available in the TCGA portal (https://cancergenome.nih.gov/). For annotation of non-promoter elements, we used HMEC Broad (GSM733705 [https://www.ncbi.nlm.nih.gov/geo/query/acc.cgi?acc=GSM733705]) and Roadmap Breast_Luminal_Epithelial (GSM669595 [https://www.ncbi.nlm.nih.gov/geo/query/acc.cgi?acc=GSM669595]) and Breast_Myoepithelial (GSM613870 [https://www.ncbi.nlm.nih.gov/geo/query/acc.cgi?acc=GSM613870]) as downloaded from the ENCODE and Roadmap browser. We used Encode Repliseq data of MCF7 cells (GSM923442 [https://www.ncbi.nlm.nih.gov/geo/query/acc.cgi?acc=GSM923442]) for time of replication (TOR) analysis. All data described within the Article are available in the Supplementary Data files and at https://github.com/tanaylab/metabric_rrbs.

Field-specific reporting

Please select the one below that is the best fit for your research. If you are not sure, read the appropriate sections before making your selection.

[x] Life sciences [ ] Behavioural & social sciences [ ] Ecological, evolutionary & environmental sciences

For a reference copy of the document with all sections, see nature.com/documents/nr-reporting-summary-flat.pdf

Life sciences study design

All studies must disclose on these points even when the disclosure is negative.

Sample size	The DNA methylation profiling (RRBS) dataset includes 1538 tumor samples and 244 adjacent normal tissues (after exclusion based on quality control). Sample size was determined by a combination of sample availability and feasibility of assay throughput. Sample size was considered sufficient to represent the breadth of disease (all subtypes) and for survival analyses.
Data exclusions	Samples with less than 1.5 million unique CpGs at a minimum 5 coverage were excluded due to insufficient data available. Samples with bisulfite conversion levels between [99.4%-99.8%] were excluded since epiclonal compositions are extremely sensitive to variations in bisulphite conversion. After exclusion, 1538 tumor samples and 244 adjacent normal tissues were retained.
Replication	Replication is not applicable since this is clinical data. The large sample size of 1538 breast tumors and 244 adjacent normal samples was considered sufficient to represent the breadth of disease (all subtypes) and for survival analyses. Validation was performed on an independent TCGA breast cancer dataset (Supplementary note 2) with highly reproducible results.
Randomization	METABRIC is a retrospective observational study so randomization was not performed. It is not relevant as the study aims to characterize features of an observational cohort.
Blinding	Blinding was not considered necessary since this is not a clinical study where patients are allocated to different treatments. In the study, all samples are processed without selection based on clinical parameters.

Reporting for specific materials, systems and methods

We require information from authors about some types of materials, experimental systems and methods used in many studies. Here, indicate whether each material, system or method listed is relevant to your study. If you are not sure if a list item applies to your research, read the appropriate section before selecting a response.

Materials & experimental systems

n/a	Involved in the study	
[x]	[ ]	Antibodies
[x]	[ ]	Eukaryotic cell lines
[x]	[ ]	Palaeontology and archaeology
[x]	[ ]	Animals and other organisms
[ ]	[x]	Human research participants
[x]	[ ]	Clinical data
[x]	[ ]	Dual use research of concern

Methods

n/a	Involved in the study	
[x]	[ ]	ChIP-seq
[x]	[ ]	Flow cytometry
[x]	[ ]	MRI-based neuroimaging

Human research participants

Policy information about studies involving human research participants

Population characteristics	The cohort includes breast cancer patients diagnosed between 1977-2005. Detailed description of the cohort can be found in Supplementary Table S1 and have been reported previously (Curtis, Nature 2012).
Recruitment	Women who were diagnosed with breast cancer between 1977-2005, underwent surgery and had available tumor tissue at one of the five tumor banks were eligible for inclusion and identified retrospectively. Full details can be found in Curtis et al. Nature 2012. There are no self selection bias in terms of sampling for METABRIC or for this particular study.
Ethics oversight	All samples were obtained with the consent from patients and appropriate approval from ethical committees (REC ref 07/H0308/161) at the University of Cambridge and the British Columbia Cancer Research Centre.

Note that full information on the approval of the study protocol must also be provided in the manuscript.
